# Genome-Wide Association Study Reveals Novel Genetic Loci for Quantitative Resistance to Septoria Tritici Blotch in Wheat (*Triticum aestivum* L.)

**DOI:** 10.3389/fpls.2021.671323

**Published:** 2021-09-24

**Authors:** Tilahun Mekonnen, Clay H. Sneller, Teklehaimanot Haileselassie, Cathrine Ziyomo, Bekele G. Abeyo, Stephen B. Goodwin, Dagnachew Lule, Kassahun Tesfaye

**Affiliations:** ^1^Institute of Biotechnology, Addis Ababa University, Addis Ababa, Ethiopia; ^2^Biosciences Eastern and Central Africa (BecA), Nairobi, Kenya; ^3^International Maize and Wheat Improvement Center- CIMMYT (Ethiopia), Addis Ababa, Ethiopia; ^4^United States Department of Agriculture (USDA)-Agricultural Research Service, West Lafayette, IN, United States; ^5^Oromia Agricultural Research Institute (OARI), Addis Ababa, Ethiopia; ^6^Ethiopian Biotechnology Institute (EBTi), Addis Ababa, Ethiopia

**Keywords:** genome-wide association study, linkage disequilibrium, population structure, quantitative trait locus, septoria tritici blotch, wheat, *Zymoseptoria titici*

## Abstract

Septoria tritici blotch, caused by the fungus *Zymoseptoria titici*, poses serious and persistent challenges to wheat cultivation in Ethiopia and worldwide. Deploying resistant cultivars is a major component of controlling septoria tritici blotch (STB). Thus, the objective of this study was to elucidate the genomic architecture of STB resistance in an association panel of 178 bread wheat genotypes. The association panel was phenotyped for STB resistance, phenology, yield, and yield-related traits in three locations for 2 years. The panel was also genotyped for single nucleotide polymorphism (SNP) markers using the genotyping-by-sequencing (GBS) method, and a total of 7,776 polymorphic SNPs were used in the subsequent analyses. Marker-trait associations were also computed using a genome association and prediction integrated tool (GAPIT). The study then found that the broad-sense heritability for STB resistance ranged from 0.58 to 0.97 and 0.72 to 0.81 at the individual and across-environment levels, respectively, indicating the presence of STB resistance alleles in the association panel. Population structure and principal component analyses detected two sub-groups with greater degrees of admixture. A linkage disequilibrium (LD) analysis in 338,125 marker pairs also detected the existence of significant (*p* ≤ 0.01) linkage in 27.6% of the marker pairs. Specifically, in all chromosomes, the LD between SNPs declined within 2.26–105.62 Mbp, with an overall mean of 31.44 Mbp. Furthermore, the association analysis identified 53 loci that were significantly (false discovery rate, FDR, <0.05) associated with STB resistance, further pointing to 33 putative quantitative trait loci (QTLs). Most of these shared similar chromosomes with already published Septoria resistance genes, which were distributed across chromosomes 1B, 1D, 2A, 2B, 2D, 3A,3 B, 3D, 4A, 5A, 5B, 6A, 7A, 7B, and 7D. However, five of the putative QTLs identified on chromosomes 1A, 5D, and 6B appeared to be novel. Dissecting the detected loci on IWGSC RefSeq Annotation v2.1 revealed the existence of disease resistance-associated genes in the identified QTL regions that are involved in plant defense responses. These putative QTLs explained 2.7–13.2% of the total phenotypic variation. Seven of the QTLs (*R*^2^ = 2.7–10.8%) for STB resistance also co-localized with marker-trait associations (MTAs) for agronomic traits. Overall, this analysis reported on putative QTLs for adult plant resistance to STB and some important agronomic traits. The reported and novel QTLs have been identified previously, indicating the potential to improve STB resistance by pyramiding QTLs by marker-assisted selection.

## Introduction

Common wheat *(Triticum aestivum* L.) is the most widely cultivated and the major staple food crop in the world consumed by human, providing almost 20% of the total calories and 21% of protein demand globally (Arzani and Ashraf, 2017; International Wheat Genome Sequencing Consortium (IWGSC), 2018; Ramadas et al., 2019). By 2050, the world's human population is projected to reach nine billion, and we will need to increase wheat production by 70% to feed this projected growth (FAO, 2009; Ray et al., 2013; Marcussen et al., 2014). Hence, boosting the wheat harvest is very pertinent to achieve zero-hunger by 2050.

Septoria tritici blotch, caused by the fungus *Zymoseptoria tritici* (anamorph: *Septoria tritici*), is an ever-existing bottleneck to wheat cultivation worldwide (Dalvand et al., 2018; Odilbekov et al., 2019), accounting for 30–54% of global wheat yield loss annually (Eyal and Levy, 1987). Septoria tritici blotch (STB) is also a major threat to wheat production in Ethiopia (Getinet et al., 1990; Takele et al., 2015; Kidane et al., 2017; Mekonnen et al., 2019, 2020), causing up to 82% of yield loss in the worst seasons (Getinet et al., 1990; Mengistu et al., 1991; Ayele et al., 2008).

The deployment of genetic resistance is the most durable, economical, and environmentally friendly method to manage crop diseases like STB (Ghaneie et al., 2011; Mekonnen et al., 2019; Odilbekov et al., 2019). In particular, qualitative and quantitative types of resistance to STB have been reported in wheat (Arraiano and Brown, 2006; Arraiano et al., 2009). The former refers to a condition where one or few major *Stb* genes provide resistance to specific *Z. tritici* isolates (Brown et al., 2015). Quantitative resistance, on the other hand, results from the expression of many genes with minor effects and is generally not specific to isolates. As such, quantitative resistance is the most effective, durable, and preferred method to manage rapidly evolving wheat pathogens such as *Z. tritici* (Long et al., 2019).

The resistance-breeding method used in Ethiopia is mainly conventional, making the crop-improvement program very slow and inefficient. Nowadays, the advent and application of modern genomic tools have revolutionized crop breeding by facilitating the precise identification, mapping, and introgression of genomic regions controlling useful agronomic traits, such as resistance, into new cultivars. To account for this, a genome-wide association study (GWAS) is a powerful approach to elucidating the genomic architecture of many traits (Long et al., 2019). The development of high-throughput sequencing and bioinformatics technologies (Huang et al., 2017) has also enabled GWAS to scan single nucleotide polymorphisms (SNPs) associated with desirable traits at the whole-genome scale (Rafalski, 2010).

Genome-wide association studies have been successfully applied to many crop species (Xiao et al., 2017) such as maize (Rashid et al., 2018), rice (Huang et al., 2017), wheat (Kidane et al., 2017; Long et al., 2019; Cheng et al., 2020), and sorghum (Girma et al., 2019). In particular, this study design has been used in wheat to analyze several traits such as resistance to stripe rust (Long et al., 2019; Yao et al., 2019; Cheng et al., 2020), stem rust (Edae et al., 2015; Kankwatsa et al., 2017), Septoria tritici blotch (Kidane et al., 2017; Odilbekov et al., 2019), drought tolerance (Mathew et al., 2019), and other phenological characteristics, plus yield and yield-related traits (Jamil et al., 2019; Wang et al., 2019; Ward et al., 2019). While Ethiopia is the largest producer of wheat in sub-Saharan Africa, little is known about the resistance Ethiopian wheat cultivars have to STB, even though it is the most important disease economically. Thus, the objectives of this study were: (1) to determine the population structure, family relatedness, and level of linkage disequilibrium of the tested bread wheat association panel; (2) to elucidate the genomic architecture of adult plant resistance to STB; (3) to identify the SNP loci underlying yield, yield-related, and phonological traits in Ethiopian cultivars that could be useful in wheat breeding programs.

## Materials and Methods

### Association Mapping Panel

This study used an association panel of 180 bread wheat (*Triticum aestivum* L.) genotypes (Supplementary Table 1), of which 167 were obtained from the International Maize and Wheat Improvement Center (CIMMYT-Mexico) and 13 were commercial cultivars grown in Ethiopia. The 167 CIMMYT genotypes included 49 from the International Bread Wheat Screening Nursery (IBWSN), 56 from the International Septoria Observation Nursery (ISEPON), 14 from the High Rain Wheat Yield Trial (HRWYT), 34 from the High Rainfall Wheat Screening Nursery (HRWSN), 5 from an adaptation trial, 6 from the National Variety Trials (NVT), and the remaining 3 genotypes were from a preliminary variety trial (PVT).

### Multi-Environment Trials

Field evaluations were carried out under natural STB infestation during the 2015 and 2016 main cropping seasons across three STB hotspots: the Holetta Agricultural Research Center (HARC) (9° 3'N/38° 30'E), Bekoji Agricultural Research Subcenter (7° 32'N/39° 15'E), and Kulumsa Agricultural Research Center (KARC) (8° 02'N/ 39° 15'E). The experimental design was an alpha lattice with two replications, six incomplete blocks, and 30 entries per sub-block per replication. The trial was sown by hand, with each entry planted in four rows of a 2.5-m length, 20-cm spacing between rows, and 40 cm between entries. The susceptible cv. “Lakech” was planted as a spreader row along the length of the blocks to create adequate disease pressure. The spaces between the blocks and replications were 1.5 m long. A seeding rate of 150 kg ha^−1^ and fertilizer rates of 100 and 75 kg ha^−1^ of N and P_2_O_5_, respectively, were used in all the experiments. Weeding was performed by hand three times each season.

### STB Evaluation

Septoria tritici blotch disease severity (SDS) was estimated visually plot-wise by considering the percentage of necrotic leaf area (NLA) on the four uppermost infected leaves of 10–20 plants (Eyal and Levy, 1987) at three growth stages, namely, heading (SDH), medium milk (SDMM), and at maturity (SDM), using a double-digit 00–99 scoring scale (Eyal and Levy, 1987). The first digit (0–9) represented blotch development in terms of plant height (for instance, 5 if the disease reached the middle (50%) of the plant height, 8 for reaching the flag leaf, and 9 for reaching the spike), while the second digit stood for the disease severity as a percentage but in terms of 0–9 (1 = 10%, 2 = 20%, and 9 = 90 %). For each stage, Septoria disease severity percentage (SDS%) was computed from the 00–99 score using the following formula as described by Sharma and Duveiller (2007):


$$\begin{array}{l} \text{SDS}=\left[\left(\text{D}1/9\right)\left(\text{D}2/9\right)\right]\text{ x }100 \end{array}$$

where D1 and D2 are the first and the second digits of the double-digit scores, respectively. The SDS values range from 0 to 100, where 0 indicates complete resistance and 100 indicates complete susceptibility (Kidane et al., 2017).

In addition, the Septoria progress coefficient (SPC) developed by Eyal and Ziv (1974) was computed to indicate the position of pycnidia relative to plant height according to the following equation:


$$\begin{array}{l} \text{SPC}=\left(\text{SDH}/\text{PH}\right) \end{array}$$

where *SDH* (Septoria disease height) is the maximum height (cm) above ground at which the pycnidia of the pathogen could be found on the plant at the maturity stage and *PH* is the average height of the genotype from the ground to the tip of its awn. The SPC coefficient indicates the position of pycnidia relative to plant height, regardless of pycnidial coverage, and allows for the comparison of the infection placements on cultivars with different plant statures. Furthermore, *SPC* values ranged from 0 to 1, where *SPC* = 0 means that there was no disease, while *SPC* =1 means that pycnidia were produced at the top of the plant (Eyal and Levy, 1987).

### Other Agronomic Data Scoring

The phenotypic data that were recorded were heading date (HD, days to 50% heading), flowering date (FD = days to 50% flowering), grain-filling duration (GFD), maturity date (MD = days to 90% maturity), grain yield, hectoliter weight (HLW = kilograms per 100 liters of wheat), thousand-kernel weight (TKW = weight of 1,000 kernels, in grams), plant height (PH), number of spikelets per spike (SPS), number of kernels per spikelet (NKPS), and number of kernels per spike (NKS). These yield data were taken from the four rows of each plot and converted to kilograms per hectare (kg ha^−1^) at 12.5% moisture content using plot size as a factor. Plant height measurement was also carried out at physiological maturity from five randomly selected and tagged plants from the middle rows of each entry.

### DNA Extraction and Genotyping by Sequencing

The wheat plants of the association panel were grown at the National Agricultural Biotechnology Research Center, Holetta under greenhouse conditions. The 2-week-old leaf samples were then collected into 96 deep-well sample collection plates, oven-dried overnight at 50°C, and sent to Integrated Genotyping Service and Support (IGSS) located at the Biosciences Eastern and Central Africa (BecA-ILRI) Hub in Nairobi, Kenya for high-density genotyping by Diversity Arrays Technology sequencing (DArTseq™ technology). Furthermore, DNA extraction was carried out using the Nucleomag Plant Genomic DNA extraction kit (Macherey-Nagel GmbH & Co. KG, Duren, Germany). Afterward, extracted DNA quality and quantity were checked on a Thermo Scientific™ NanoDrop™ 2000 Spectrophotometers (Thermo Scientific™, USA) and on 0.8% agarose gels. As a result, the extracted genomic DNA concentration ranged from 50 to 100 ng/μl. Whole-genome profiling was also carried out using the genotyping-by-sequencing (GBS) platform as described by Elshire et al. (2011). This method involved library construction following the DArTSeq complexity reduction method *via* the digestion of genomic DNA using *ApeKI* [a type II restriction endonuclease that recognizes a degenerate 5-bp sequence (GCWGC, where W is A or T)] and the ligation of barcoded adapters, which was also followed by the PCR amplification of adapter-ligated fragments. The libraries were then sequenced using single-read sequencing runs for 77 bases. The next-generation sequencing of the GBS library was also carried out using an Illumina HiSeq 2500 lane (Illumina, San Diego, CA, United States) following the protocol of the manufacturer.

### Quality Control and SNP Calling

The technical quality of the sequencing was checked using a Sequencing Analysis Viewer. DArTSeq markers were scored using the *DArTsoft*14 software implemented in the KDCompute plug-in system developed by Diversity Arrays Technology (2017) (http://www.kddart.org/kdcompute.html) based on their alignment with the reference genome of the Chinese Spring Wheat RefSeq v1.0 [International Wheat Genome Sequencing Consortium (IWGSC), 2018], which was obtained from the International Wheat Genome Sequencing Consortium database (https://urgi.versailles.inra.fr/download/iwgsc/) at a minimum base identity of 90% and e-value of 5e-10. Two types of markers were scored, namely, SilicoDArT markers and SNP markers, which were both scored in a binary fashion (1/0), indicating the presence or absence of a marker in the genomic representation of each sample as described by Akbari et al. (2006). Marker quality was also maintained by removing monomorphic markers and those with lower call rates (>30% missing) and MAFs (minor allele frequencies) <5% using the ArTSoft14 software.

### Statistical Data Analysis

#### Phenotypic Data Analysis

We conducted an ANOVA for each location in each year using the SAS software version 9.2 (SAS Institute Inc., 2008) by considering genotype and the block as fixed and random factors, respectively. In an individual environment, the observed phenotypic response of the ith genotype in the jth replication and lth sub-block was computed using the following model:


(1) $$\begin{array}{r} y_{ijl}=\mu +g_{i}+\gamma_{j}+bl_{\left(j\right)}+\epsilon_{ij} \end{array}$$

where y_ijl_ = the observed phenotype, μ = the grand mean, g_i_ = fixed effect of the i^th^ genotype, γ_j_ = effect of the j^th^ replication, bl_(j)_ = random effect of the l^th^ block nested within the j^th^ replication, and ε_ijl_ = random error term.

The ANOVA of all seasons and locations was executed by considering genotype as a fixed effect and the block, location, and year as random effects according to the following model:


$$\begin{array}{l} {\text{Y}}_{ijklm}=\mu +{\text{g}}_{m}+\gamma_{\text{ijk}}+{\text{y}}_{\text{ij}}+{\text{e}}_{\text{j}}+{\text{b}}_{\text{ijkl}}+{\left(\text{gy}\right)}_{\text{im}}\\ +{\left(\text{ge}\right)}_{\text{jm}}+{\left(\text{ye}\right)}_{\text{ij}}+{\left(\text{yeg}\right)}_{\text{ijm}}+\epsilon_{\text{ijklm}} \end{array}$$

where Y_ijklm_ = observed response of genotype m, replication k of block l of location j and year i; μ = grand mean; g_m_ = fixed effect of genotype m; r_ijk_ = effect of replication k in location j and year i; y_ij_ = random effect of year i at location j that is ~ normally and independently distributed (NID) (0, $\delta_{\text{y}}^{2}$); e_j_ = random effect of location j that is ~ NID (0, $\delta_{\text{e}}^{2}$); b_ijkl_ = random effect of block l nested with replication k in location j and year i that is ~ NID (0, $\delta_{\text{b}}^{2}$); (gy)_im_ = random effect of the interaction between genotype m and year i that is ~ NID (0, $\delta_{\text{gy}}^{2}$); (ge)_jm_ = random effect of the interaction between genotype m and location j that is ~ NID (0, $\delta_{\text{ge}}^{2}$); (ye)_ij_ = random effect of the interaction between year i and location j that is ~ NID (0, $\delta_{\text{ye}}^{2}$); (yeg)_ijm_ = random effect of the three-way interaction of genotype m in location j and year i that is ~ NID (0, $\delta_{\text{gey}}^{2}$); ε_ijklm_= random residual effect that is ~ NID (0, $\delta_{\varepsilon }^{2}$).

The variance components were also computed. The broad-sense heritability (H^2^) within an environment was estimated for the traits from an ANOVA using the following formula:


$$\begin{array}{l} H^{2}=\left(\delta^{2}g\right)/\left(\delta^{2}g+\delta^{2}\epsilon /r\right) \end{array}$$

The broad-sense heritabilities across the environments were also estimated by the formula:


$$\begin{array}{l} H^{2}=(\delta^{2}g)/(\delta^{2}g+\delta^{2}gy/y+\delta^{2}ge/l+\delta^{2}gye/yl+\delta^{2}\epsilon /ylr) \end{array}$$

where δ^2^ g is the genotypic variance, σ^2^gy is the genotype-by-year interaction variance, σ^2^ge is the genotype-by-location interaction variance, σ^2^gye is the genotype-by-year-by-location interaction variance, δ^2^e is the location variance, and l, r, and y represent the numbers of locations, replicates, and years, respectively. The percentage of heritability was categorized as low (<30%), moderate (30–60%), or high (≥60%) as described by Robinson et al. (1949). The relationship between agronomic traits was also determined by Pearson's correlation using the SAS software version 9.2 (SAS Institute Inc., 2008).

#### Population Structure Analysis

A population stratification of the association panel was visualized by principal component analysis (PCA) using the KDCompute plug in system version 1.0.1 (https://kdcompute.seqart.net/kdcompute/plugins). Population admixture patterns were also determined using a Bayesian model-based clustering algorithm implemented in the STRUCTURE software v.2.3.4 (Pritchard et al., 2000). The STRUCTURE program was run with the admixture model, correlated allele frequencies, a burn-in period of 10,000, and 50,000 Markov Chain Monte Carlo (MCMC) replications after a burn in for hypothetical subpopulations K from 1 to 10 with 10 iterations. The optimum K value was predicted based on a study by Evanno et al. (2005) using STRUCTURE HARVESTER ver. 0.6.92 (Earl and von Holdt, 2012). A bar plot for the optimum K was determined using Clumpak beta version (Kopelman et al., 2015).

### Genome-Wide Association Study

The association mapping of phenotypic traits with genome-wide scanned SNPs was conducted using the Genome Association and Prediction Integrated Tools (GAPIT) package (Lipka et al., 2012) in the R software (R Core Team, 2013). This GWAS was carried out for four Septoria disease traits, namely, SDSH, SDSMM, SDSM, and SPC, and some important agronomic traits such as the days to 50% heading (DH), days to 50% flowering (DF), grain filling duration (GFD), days to 90% maturity (DM), grain yield, thousand-kernel weight (TKW), and plant height (PH) in each individual environment; the study design also used the means across all environments [the best linear unbiased estimate (BLUE) values]. The analysis involved a total of 7,776 robust SNPs with a call rate of >70% and MAF of >5%. Missing SNP data were imputed using optimal impute ver. 1.0.0 in the KDcompute_plugin system based on the KNN imputation method. The marker distribution on each chromosome was determined using LD measure in *R*^2^ ver.0.2.2 of the KDcompute_plugin. Pairwise LD measures (*r*^2^ and *P*-value) between markers on each chromosome were also computed using TASSEL Ver. 5 (Bradbury et al., 2007). A genome-wide LD decay scatter plot was then produced by plotting the *r*^2^ values against physical distance (bp) using the GAPIT software. Finally, *r*^2^ = 0.2 was considered as a cutoff point for no LD between pairs of markers.

The GWAS was conducted using the fixed and random model circulating probability unification (FarmCPU) algorithm (Liu et al., 2016) implemented in the GAPIT R package (2.0) (Tang et al., 2016). The algorithm uses both fixed-effect and random-effect models iteratively to control spurious marker-trait associations due to population structure and family relatedness (Lipka et al., 2012). Furthermore, a kinship (K) matrix was computed using the method of VanRaden (2008). Principal components describing the population stratification were computed using R/GAPIT and iteratively added to the fixed part of the model. Quantile–quantile (Q–Q) plots generated from –log10 *p*-values were assessed visually to determine how well the model accounted for population structure and family relatedness among the study samples. Statistically significant marker-trait associations were declared using a false discovery rate (FDR)-adjusted *p* < 0.05 as implemented in GAPIT. Furthermore, the Bonferroni correction rate at a significance threshold of *p* < *0.1*5 or –log10 (*p-*values) = 4.71 was also included in the analysis for comparison. Both the Q–Q and Manhattan plots were visualized using the R package qqman (Turner, 2014). The high-confidence candidate genes within the identified resistance-associated regions were also extracted from the recently released IWGSC RefSeq Annotation v2.1 available on the URGI Seq repository (https://wheat-urgi.versailles.inrae.fr/Seq-Repository/Annotations).

## Results

### Phenotypic Data Analysis

#### Adult Plant Responses to STB and Broad-Sense Heritability

**Figure 1:**
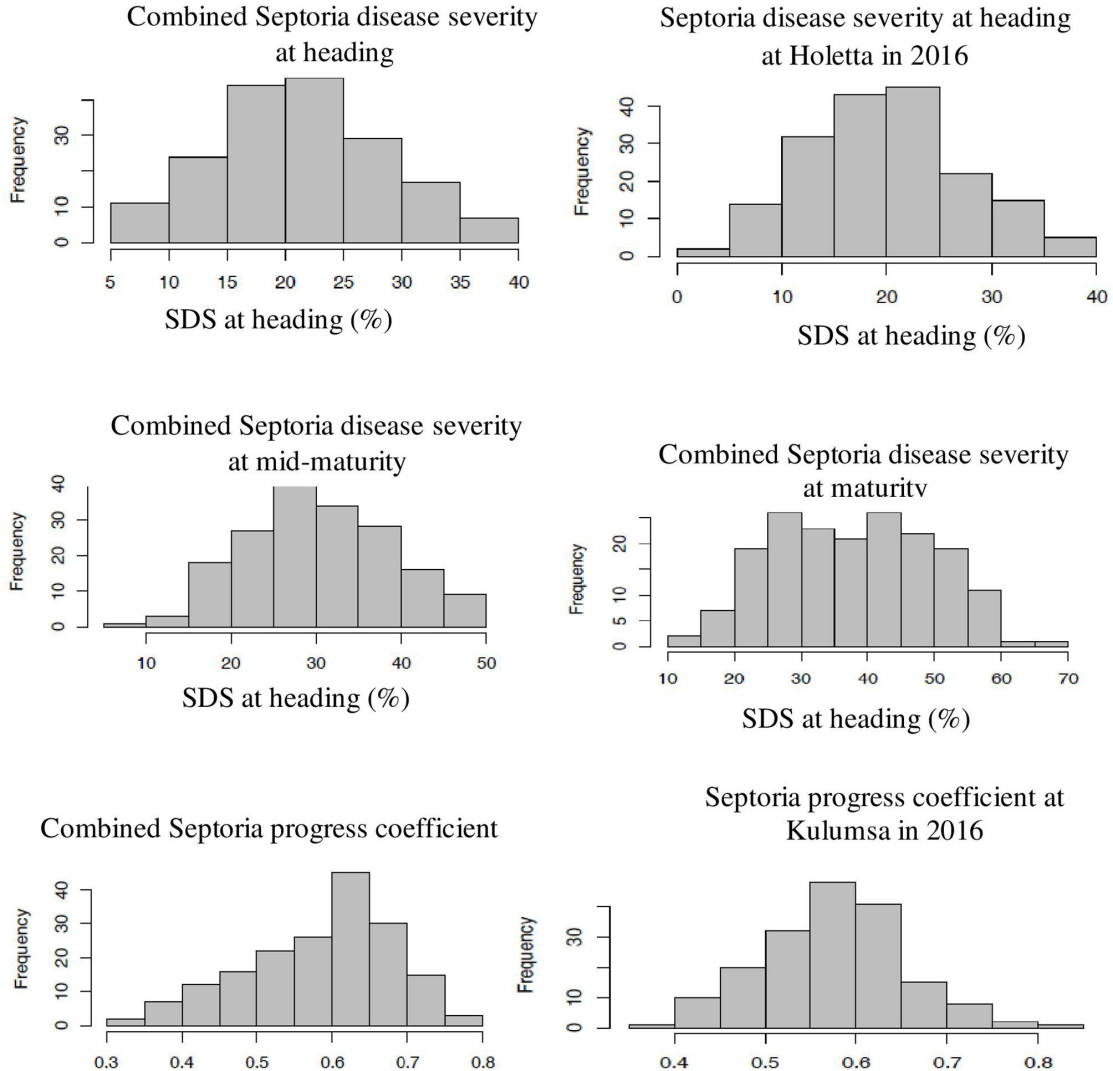
Frequency distribution of some SDS traits. The combined data were from three locations and over 2 years field evaluation of 180 wheat genotypes. The right and left ends of the graphs indicate the highest and lowest affection classes, respectively. The combined Septoria disease severity at heading, mid-maturity, and maturity stages followed a normal distribution. The severity values increased from heading to mid-maturity, and then to maturity stages. Combined Septoria progress coefficient showed pseudo-normal distribution, confirming the quantitative nature of STB resistance in the tested wheat material. The x-axis represents the BLUE value of the study genotype.

The genotype effect was significant (*p* < *0.0*001) for STB resistance at all the growth stages in all the test environments. Genotypic variance (σ^2^g) was the major contributor to STB resistance variability among the tested wheat genotypes. The Septoria disease severity traits also showed pseudo-normal distributions (Figure 1), indicating the quantitative nature of STB resistance in the tested wheat genotypes (Kidane et al., 2017). The analysis revealed that the STB infestation showed seasonal fluctuations, but that was still higher during the 2015 growing season (Table 1). Moreover, the disease severity showed an increasing trend from heading to the maturity stage. In each environment, mean SDS values at the heading and mid-maturity stages ranged from 18.2 to 31.2% and 21.7 to 37.6%, respectively, while the highest severity values were registered at Holetta in 2015. The mean disease severity at maturity and its vertical progress varied from 30 to 50.8% and 0.41 to 0.69, respectively, while they were the highest at Bekoji in the 2015 growing season. The lowest Septoria severity was recorded at Kulumsa in 2016. The broad-sense heritability for Septoria resistance in each environment ranged from moderate (H^2^ = 0.58) to high (H^2^ = 0.99) (Table 2).

**Table 1:** Descriptive statistics of SDS values in bread wheat genotypes evaluated in three locations in Ethiopia during the 2015 and 2016 growing seasons.

**Trait**	**Environment**	**Mean**	**Range**	**SD**	**Pr > F**	**Trait**	**Environment**	**Mean**	**Range**	**SD**	**Pr > F**
SDSH	E1 (HARC2015)	31.23	72.84	8.88	^***^	SDSM	E1 (HARC 2015)	46.9	95.68	24.07	^***^
	E2 (Bekoji 2015)	24.48	63.58	6.8	^***^		E2 (Bekoji 2015)	50.77	77.16	19.05	^***^
	E3 (Kulumsa 2015)	23.47	40.74	8.00	^***^		E3 (Kulumsa 2015)	32.38	70.99	9.44	^***^
	E4 (HARC 2016)	20.09	39.81	4.88	^***^		E4 (HARC 2016)	33.07	65.44	1.8	^***^
	E5 (Bekoji 2016)	19.36	44.71	9.08	^***^		E5 (Bekoji 2016)	34.28	55.56	10.41	^***^
	E6 (Kulumsa 2016)	18.16	38.89	6.87	^***^		E6 (Kulumsa 2016)	30.04	46.94	9.61	^***^
SDSMM	E1 (HARC 2015)	37.6	82.72	22.7	^***^	SPC	E1 (HARC 2015)	0.65	0.87	0.2	^***^
	E2 (Bekoji 2015)	34.56	74.07	17.2	^***^		E2 (Bekoji 2015)	0.69	0.74	0.12	^***^
	E3 (Kulumsa 2015)	32.13	49.51	8.2	^***^		E3 (Kulumsa 2015)	0.41	0.60	0.09	^***^
	E4 (HARC 2016)	27.13	58.95	5.29	^***^		E4 (HARC 2016)	0.6	0.62	0.09	^***^
	E5 (Bekoji 2016)	25.6	47.03	9.51	^***^		E5 (Bekoji 2016)	0.58	0.61	0.12	^***^
	E6 (Kulumsa 2016)	21.73	39.51	8.35	^***^		E6 (Kulumsa 2016)	0.58	0.46	0.08	^***^

*SDSH, Septoria disease severity at heading; SDSMM, Septoria disease severity at mid- maturity; SDSM, Septoria disease severity at maturity; SPC, Septoria progress coefficient; SD, standard deviation*;

*** *, very highly significant (p ≤ 0.0001); “sn”, non-significant at the α, 5% significance level*.

**Table 2:** Genotypic variance (σ^2^g) and heritability in the broad sense (H^2^) for phenotypic traits in 180 bread wheat genotypes in six different environments in Ethiopia.

	**Holetta-2015 (E1)**		**Bekoji-2015 (E2)**		**Kulumsa-2015 (E3)**		**Holetta-2016 (E4)**		**Bekoji-2016 (E5)**		**Kulumsa-2016 (E6)**	
**Trait**	**σ^2^g**	**H^**2**^**	**σ^2^g**	**H^**2**^**	**σ^2^g**	**H^**2**^**	**σ^2^g**	**H^**2**^**	**σ^2^g**	**H^**2**^**	**σ^2^g**	**H^**2**^**
SDSH	344.51^***^	0.90	212.66^***^	0.90	39.14^***^	0.61	42.56^***^	0.78	79.41^***^	0.96	44.67^***^	0.95
SDSMM	485.36^***^	0.94	255.28^***^	0.86	38.7^***^	0.58	92.23^***^	0.90	88.63^***^	0.98	66.48^***^	0.95
SDSM	516.40^***^	0.89	275.44^***^	0.76	331.16^***^	0.88	127.99^***^	0.99	105.24^***^	0.97	86.59^***^	0.94
SPC	0.04^***^	0.88	0.01^***^	0.70	0.02^***^	0.87	0.01^***^	0.62	0.01^***^	0.83	0.00^***^	0.67

*σ^2^g, genotypic variance; H^2^, heritability in the broad sense (H^2^)*;

*** *, very highly significant (p ≤ 0.0001); “sn” = non-significant at the α, 5% significance level*.

The combined ANOVA revealed that the effect of genotype, year, location, and their two- (genotype × year, genotype × location, and year × location) and three-way interactions (genotype × year × location) were significant for SDS traits except for the Septoria progress coefficient, where the effect of year was not significant (Table 3). The analysis of pooled data revealed that genotypic variance (σ^2^g) was the highest for all the SDS parameters except SDSMM, where environmental effect was the highest (Table 4). The broad-sense heritabilities of Septoria resistance traits showed that they were highly heritable (H^2^ = 72– 81%) (Table 4) (Robinson et al., 1949). The phenotypic and genotypic coefficients of variation for SDS traits ranged from 32.4 (SPC) to 68.3% (SDSH) and 22.5 (SPC) to 41.4% (SDSM), respectively. At 5% selection intensity, the genetic advance for SDS traits ranged from 0.31 (SPC) to 35.23 (SDSM), while the magnitude of the expected genetic gains as a percent of the mean varied from 53.69% (SPC) to 102.38% for SDSH (Table 4).

**Table 3:** Combined analysis of variance for Septoria disease severity traits across three locations in Ethiopia over years 2015 and 2016.

**Source of variation**	**DF**	**Mean squares**			
		**SDSH**	**SDSMM**	**SDSM**	**SPC**
Genotype	179	699.53^***^	874.84^***^	1650.29^***^	0.12^***^
Replication	1	662.48^*^	35.41^ns^	5498.56^***^	0.87^***^
Year	1	29489.27^***^	44295.09^***^	64059.86^***^	0.01^ns^
Location	2	5257.27^***^	2734.03 ^***^	24849.55^***^	4.57^***^
Incomplete block (nested)	5	1468.79^***^	2167.8^***^	2955.71^***^	0.31^***^
Genotype*year	179	310.41^***^	392.85^***^	1008.41^***^	0.06^***^
Genotype*location	358	207.74^***^	288.83^***^	172.91^***^	0.02^***^
Year*location	2	1987.73^***^	358.46^ns^	10452.82^***^	3.78^***^
Genotype*Year*Location	358	148.97^***^	213.74^***^	152.76^*^	0.02^***^

*SDSH, Septoria disease severity at heading; SDSMM, Septoria disease severity at mid-maturity stage; SDSM, Septoria disease severity at maturity; SPC, Septoria progress coefficient*;

*** *, very highly significant at p <0.0001*;

* *, significant at p < 0.05*;

*ns, non-significant at the p = 0.05 significance level; DFs, degrees of freedom*.

**Table 4:** Variance component estimates for SDS, H^2^ (broad sense), genotypic coefficient of variance (GCV), phenotypic coefficient of variance (PCV), genetic advance (GA), and genetic advance as percent of the mean (GAM) based on pooled data from the six environments.

**Trait**	**σ^2^g**	**σ^2^gy**	**σ^2^gl**	**σ^2^gyl**	**σ^2^e**	**H^**2**^**	**GCV**	**PCV**	**GA**	**GAM**
SDSH	81.97^***^	26.91^***^	14.7^***^	34.63^***^	79.71^***^	0.73	40.07	68.26	23.14	102.38
SDSMM	97.67^***^	29.86^***^	18.78^***^	40.46^***^	132.82^***^	0.72	32.62	59.01	26.36	86.99
SDSM	246.24^***^	142.61^***^	5.04^***^	15.32^***^	122.13^***^	0.75	41.41	60.83	35.23	93.09
SPC	0.02^***^	0.01^***^	−0.01^***^	0.01^***^	0.01^***^	0.81	22.51	32.37	0.31	53.69

*σ^2^g, genotypic variance estimate; σ^2^gL, genotype × year interaction variance estimate; σ^2^gL, genotype × location interaction variance estimate; σ^2^gyl, genotype × year × location interactions variance estimate; σ^2^e, residual variance estimate*;

*** , very highly significant at p < 0.0001; ^*^, significant at p < 0.05; ns, non-significant at the p = 0.05 significance level; SDSH, Septoria disease severity at heading; SDSMM, Septoria disease severity at mid maturity stage; SDSM, Septoria disease severity at maturity; SPC, Septoria progress coefficient.

Over all the environments, the average SDS of the individual wheat genotypes ranged from 5.3 to 39.8% at heading, 8.2 to 48.5% at mid-maturity, and 10.6 to 65.3% at maturity (Supplementary Table 2). The average SPC of the individual environments ranged from 0.37 to 0.79. The most resistant genotype at all the growth stages was G174, while G104 (39.8%), G76 (48.5%), and G127 (65.3%) were the most susceptible genotypes at the heading, mid-maturity, and maturity stages, respectively (Supplementary Table 2). A comparative severity analysis with the standard checkKing-bird (G40) and the mean performance of the released varieties also confirmed the presence of superior STB-resistant genotypes among the tested materials. Of the 180 tested genotypes, 56 (31%) at heading, 75 (42%) at mid-maturity, and 105 (59%) at maturity had numerically superior STB resistance compared with King-bird (Supplementary Table 2).

The top 5% best genotypes at maturity had 47.6–71% greater resistances than King-bird and 11.9–74.4 % greater resistances compared with the mean performances of the released varieties (Table 5).

**Table 5:** Comparison of the mean performances of 5% of the genotypes selected for Septoria tritici blotch (STB) resistance with King-bird, a recently released variety, and with the mean performances of 13 released varieties.

		**Comparative advantage for STB resistance (% over)**				**Comparative advantage for STB resistance (% over)**	
**Genotypes**	**Mean of selected genotypes**	**King-bird**	MRV^*^	**Genotypes**	**Mean of selected genotypes**	**King-bird**	MRV^*^
**Septoria disease severity at heading (%)**				**Septoria disease severity at maturity (%)**			
G174	5.25	70.95	74.4	G174	10.6	74.25	71.83
G153	5.82	67.81	71.63	G144	14.21	65.49	62.24
G144	5.87	67.53	71.38	G3	15.23	63	59.51
G150	6.07	66.39	70.38	G153	15.95	61.25	57.6
G151	8.45	53.22	58.78	G156	18.73	54.5	50.21
G141	8.9	50.72	56.57	G133	18.83	54.25	49.94
G133	8.9	50.72	56.57	G151	19.04	53.75	49.4
G3	9.32	48.44	54.56	G155	19.45	52.75	48.3
G156	9.47	47.58	53.81	G97	19.66	52.25	47.75
King-bird	18.06	–	11.88	King-bird	41.15	–	−9.43
MRV^*^	20.49	13.48	–	MRV^*^	37.61	8.62	–
**Septoria disease severity at mid-maturity (%)**				**Septoria progress coefficient**			
G174	8.18	70.4	72.74	G174	0.31	50.44	45.59
G153	10.55	61.83	64.86	G144	0.35	44.51	39.09
G144	14.15	48.79	52.85	G133	0.37	40.82	35.03
G3	14.46	47.68	51.83	G3	0.37	40.31	34.48
G151	15.28	44.7	49.08	G155	0.37	40.3	34.46
G155	15.75	43.02	47.54	G151	0.38	38.97	33.01
G92	16.31	40.97	45.65	G154	0.39	38.03	31.97
G150	16.36	40.79	45.48	G97	0.39	37.76	31.68
G81	17.03	38.37	43.25	G47	0.4	36.88	30.71
King-bird	27.63	–	7.93	King-bird	0.62	–	9.78
MRV^*^	30.01	−8.61	–	MRV^*^	0.57	8.91	–

* *MRV, mean of 13 selected released varieties. Negative values for comparative advantage indicate less STB resistance (inferior performance) of the genotype*.

Pearson's correlation analysis of the means over all environments revealed that STB resistance traits were significantly negatively associated with important agronomic traits. Except for SPC, all the SDS traits showed non-significant and negligible negative correlations (*r* < −0.3) with plant height. Disease traits also showed little to negative associations with HD, FD, GFD, NKPS, and NKS. However, a significant weak negative association (−0.25 to −0.48) was observed between SDS traits and MD, grain yield, HLW, and TKW (Table 6).

**Table 6:** Correlation analyses among Septoria resistance traits and some agronomic traits in 180 bread wheat genotypes based on the pooled data from 2 years of field trials in Ethiopia.

	**HD**	**FD**	**MD**	**GFD**	**Yield**	**HLW**	**TKW**	**PH**	**NKPS**	**NKS**
SDSH	−0.04^ns^	−0.05^ns^	−0.19^*^	−0.21^*^	−0.43^***^	−0.32^***^	−0.42^***^	−0.1^ns^	−0.19^*^	−0.19^*^
SDSMM	−0.1^ns^	−0.1^ns^	−0.25^*^	−0.24^**^	−0.48^***^	−0.33^***^	−0.51^***^	−0.08^ns^	−0.25^**^	−0.21^*^
SDSM	−0.21^**^	−0.22^*^	−0.35^***^	−0.26^**^	−0.47^***^	−0.32^***^	−0.44^***^	−0.06^ns^	−0.16^*^	−0.14^ns^
SPC	−0.15^*^	−0.14^ns^	−0.29^***^	−0.22^*^	−0.38^***^	−0.25^**^	−0.44^***^	−0.21^*^	−0.12^ns^	−0.1^ns^

*HD, heading date; FD, flowering date; MD, maturity date; GFD, grain-filling duration; HLW, hectoliter weight; TKW, thousand-kernel weight; PH, plant height; NKPS, number of kernels per spikelet; NKS, number of kernels per spike; SDSH, Septoria disease severity at heading; SDSMM, Septoria disease severity at mid maturity stage; SDSM, Septoria disease severity at maturity; SPC, Septoria progress coefficient*.

*** *, very highly significant (p < 0.0001)*;

** *= highly significant*;

* *, significant*;

*ns, non-significant at the α, 5% significance level; (–), negative correlation. The magnitude of the correlation coefficient indicates the strength of the correlation*.

#### SNP Statistics

The Illumina HiSeq 2500 (Illumina, San Diego, CA, United States) sequencing failed to generate SNP data for two genotypes (9 and 95); hence, a total of 178 bread wheat genotypes were successfully DArTSeq genotyped. Initially, a total of 35, 672 SNPs were discovered, of which 31,052 (87%) were mapped to known chromosomal positions on the reference used and 828 (2%) of the sequences were mapped to unknown chromosomes in the reference. In contrast, approximately 11% (3,792) of the SNPs did not align to any of the chromosomes of the wheat reference genome. Furthermore, the discovered DArTSeq SNPs were not evenly distributed among the sub-genomes, with the A, B, and D genomes accounting for 10,317, 10,979, and 9,756 SNPs, respectively (Supplementary Figure 1). Among the 21 wheat chromosomes, the highest (2,065) and the lowest (833) numbers of SNPs were mapped to chromosomes 7D and 4D, respectively (Supplementary Figure 1), and on average, each chromosome harbored about 1,479 SNPs. Maintaining SNPs with higher call rate (>70%) and MAF >0.05 resulted in 7,776 SNP markers, among which 87.3% had a known chromosome position in the wheat reference genome. Among the filtered SNPs, 2,410 were distributed on the A genome, 2,872 were distributed on the B genome, and 1,506 were distributed on the D genome. The remaining 988 SNPs were assigned to a hypothetical chromosome “0” for the sake of analysis. Hence, 7,776 SNPs were used in downstream analyses, which included principal component analysis (PCA), population clustering, population structure, LD, and GWAS.

### Population Structure Analysis

**Figure 2:**
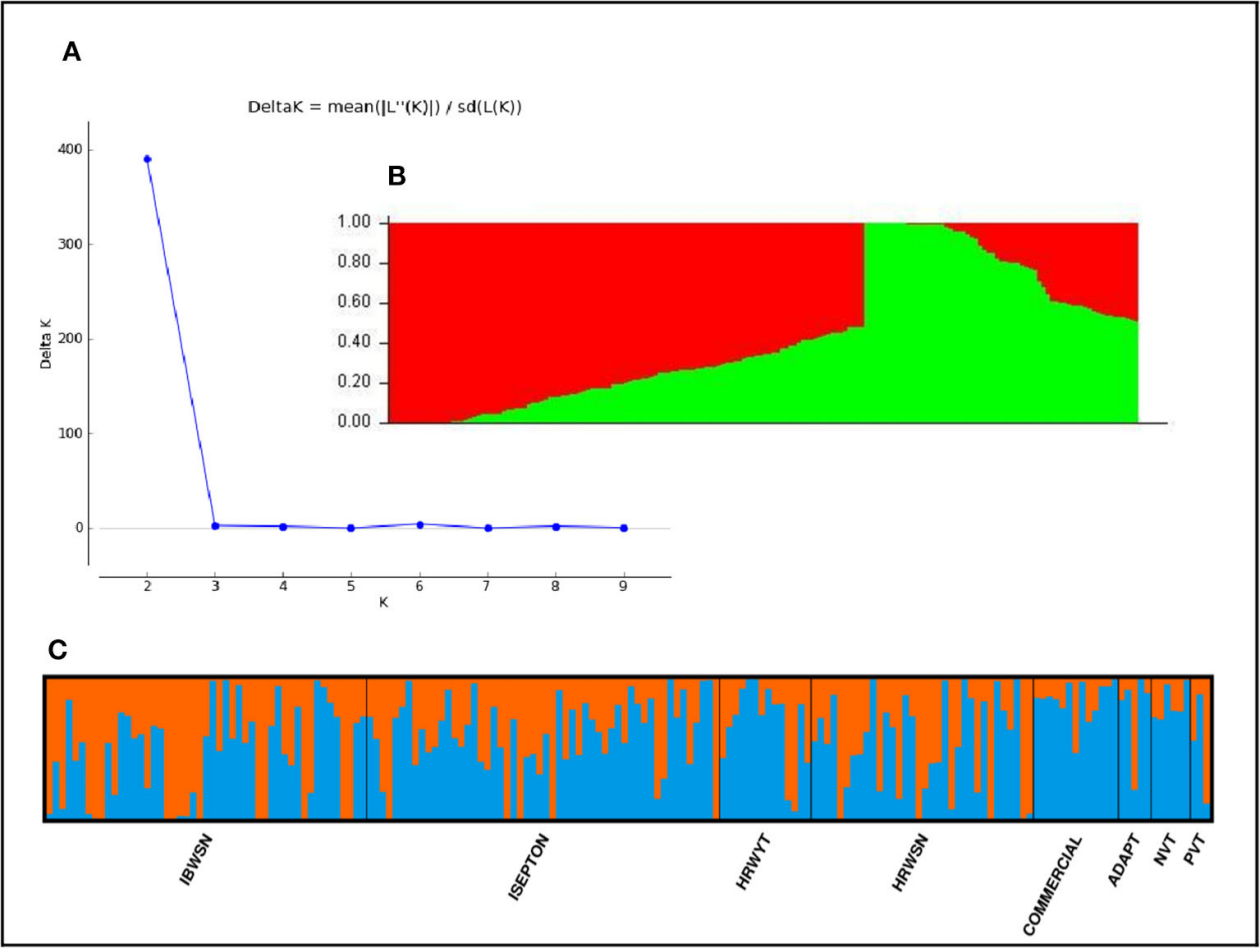
Population structure of the 178 bread wheat genotypes representing eight populations. **(A)** Best delta K value estimated using the method of Evanno et al. (2005), and the pick at k = 2 indicates the number of subpopulations in our collection, **(B)** Population structure plot and SP1 and SP2 represents subpopulations 1 and 2, respectively, **(C)** Estimated population structure for K = 2 according to the breeding materials. The different (blue and orange) co lures represent genetic groups or sub-populations designated by Structure Harvester: the x-axis represents individual samples and y-axis represents the proportion of ancestry to each cluster. Population abbreviations are: IBWSN, International Bread Wheat Screening Nursery; ISEPTON, International Septoria Observation Nursery; HRWYT, High Rain Wheat Yield Trial; HRWSN, High Rain Wheat Screening Nursery; ADAPT, Adaptation t1ial; NVT, National Verification Trial, and PVT, Preliminary Verification.

**Figure 3:**
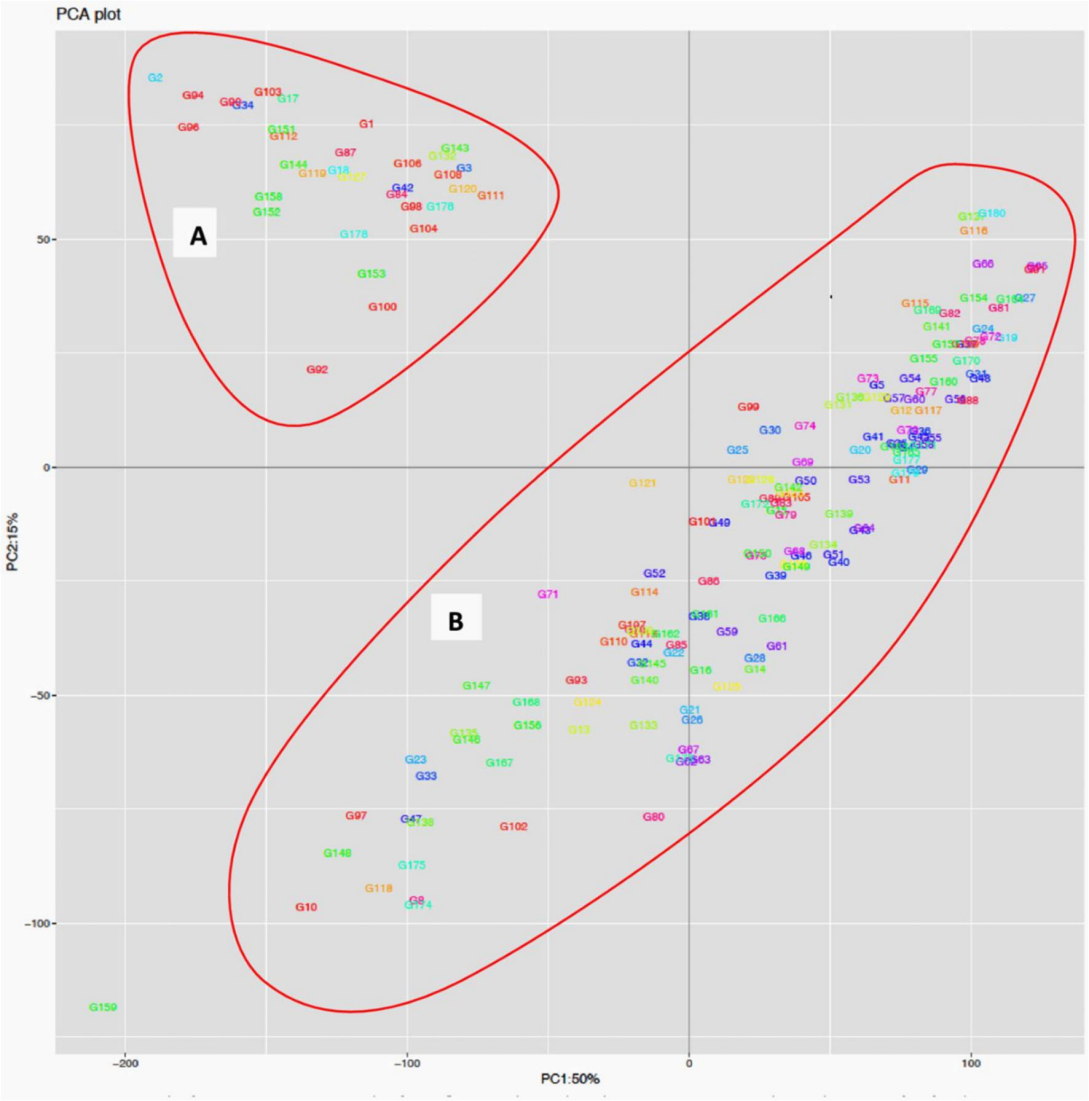
Population structure analysis of the 178 bread wheat genotypes based on principal component analysis clustering as revealed by the first two principal components. Samples coded with the same color belong to same population. Cluster **(A)** composed of 33 (18.54%) genotypes while Cluster **(B)** possessed 145 (81.46%) of the genotypes.

**Figure 4:**
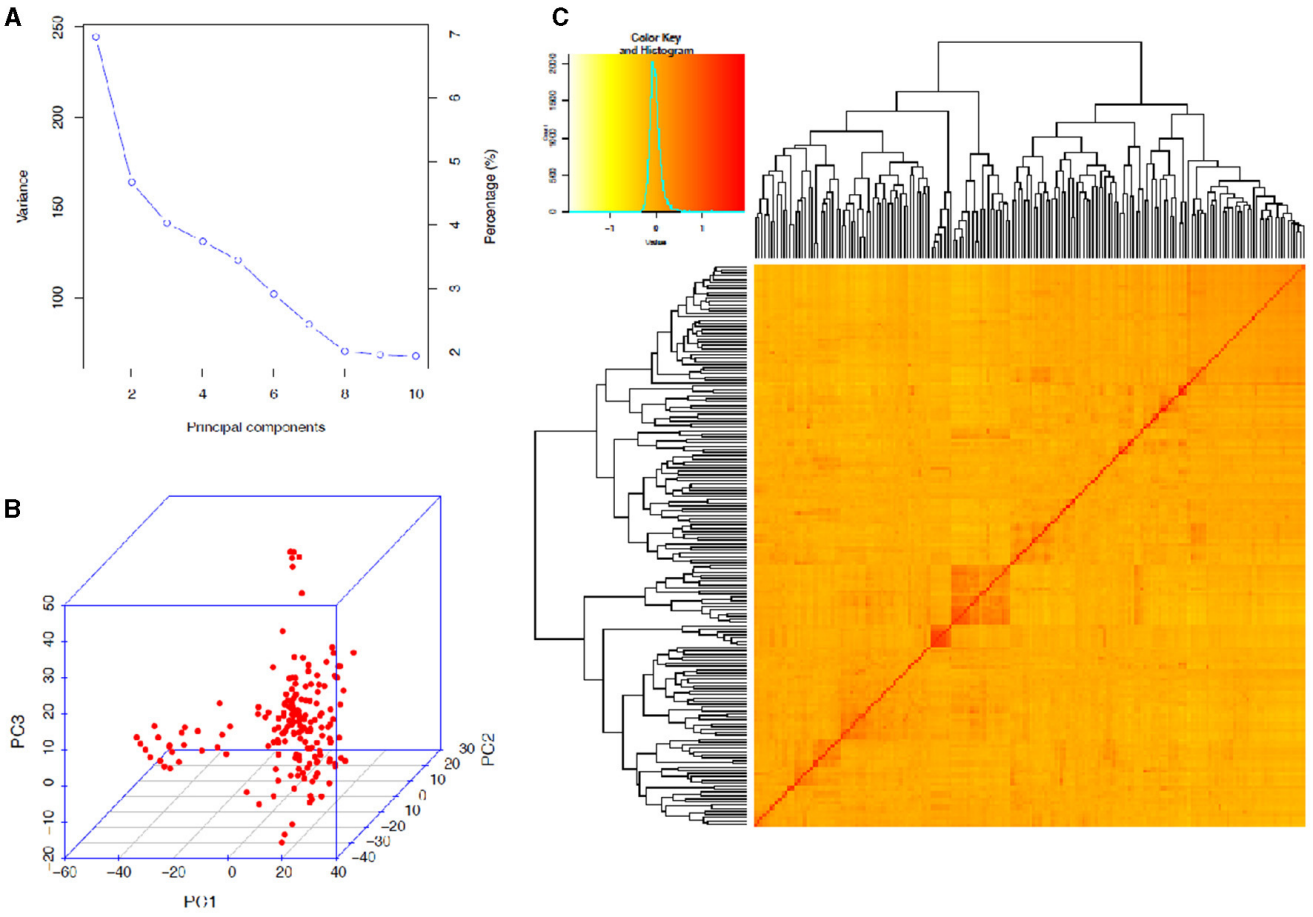
Principal component and familiar relatedness analyses of 178 bread wheat genotypes based on 7,776 genome-wide scanned, high-quality SNPs. **(A)** A screen plot displaying the first 10 principal components with their corresponding fraction of variation explained, **(B)** 3D plots of the first three principal components to depict the samples' relationship in space, and **(C)** Heat map showing the kinship analysis. The kinship values showed a normal distribution (turquoise curve), and orange and red colors represent weak and high kinship relations in the panel, respectively. The resulted clustering tree is indicated outside of the matrix.

The STRUCTURE analysis indicated two sub-populations in the association panel (Figure 2A), where ~43% (76) of the genotypes were assigned to cluster one and 57% (101) were assigned to cluster two. Additionally, the Clumpak result detected a greater degree of genetic admixture between the two sub-populations (Figure 2B), where all the individual genotypes shared alleles inherited from both subgroups (Figure 2C), thus confirming the presence of close relationships among the study materials. Furthermore, the PCA results also suggested the presence of two sub-populations (Figure 3). The first two principal components explained 65% (PC1 = 50% and PC2 = 15%) of the total variance contained in the data (Figure 3). With this, a scree plot, which was used to display the proportion of variation captured by each of the 10 principal components, also showed that the first two principal components (PC1 and PC2) explained the highest proportion of the total variation in the panel (Figure 4A). Figure 4B represents the 3D plots of the first three principal components to depict the samples' relationship in space, the analysis also confirmed the presence of kinship in the association panel (Figure 4C), suggesting the importance of using a powerful statistical GWAS model that accounts for the population structure and familial relatedness in the association study.

#### Linkage Disequilibrium (LD) Analysis

The linkage disequilibrium of alleles at different loci varied considerably across each chromosome and among chromosomes and sub-genomes (Table 7). There was a total of 338,125 marker pairs with average LD values of *r*^2^ = 0.11, with 97,723 (27.6%) pairs showing significant linkage at *p* ≤ 0.01 (Table 7). In particular, the B genome harbored the highest (143,600 or 42.5%) number of marker pairs, followed by the A genome with 119,225 (35.5%) of the marker pairs (Table 7). In contrast, the D genome harbored the lowest number (75,300 or 22.3%) of the marker pairs. Relatively, however, the SNPs on the B genome showed the strongest LD, with a mean value of *r*^2^ = 0.1187. Over all the chromosomes, the LD between SNPs declined to *r*^2^ = 0.2 within a physical distance of 31.44 Mbp; this ranged from 2.26 to 105.6 Mbp by chromosome. The weakest and strongest LD values were observed between the marker pairs on chromosomes 4D (*r*^2^ = 0.0251) and 2D (*r*^2^ = 0.211), respectively (Table 7). The physical distance (bp) at which the LD decayed to the critical *r*^2^ (0.2) value was used to determine the confidence interval for declaring the distinct QTL for each chromosome. Significant SNP markers from the same chromosome were also assigned to the same QTL if the distance between the significant markers was less than the critical physical distance.

**Table 7:** Summary of linkage disequilibrium analyses among marker pairs and the number of significant marker pairs per chromosome and genome.

**Chromosome**	**TNMP**	**r^**2**^**	**Distance (Mbp)**	**Significant marker pairs (*P* < 0.01)**
1A	12,475	0.13	58.05	4,374 (35.06)
1B	19,900	0.10	44.93	5,910(29.7)
1D	10,250	0.128	57.08	1,783 (17.40)
2A	21,200	0.15	48.03	7,342 (34.63)
2B	28,100	0.11	36.88	9,511 (33.85)
2D	14,550	0.21	57.45	4,771 (32.79)
3A	17,450	0.09	57.38	4,259 (24.41)
3B	22,000	0.12	49.85	6,795 (30.89)
3D	14,650	0.12	56.41	3,940 (26.90)
4A	13,050	0.11	75.78	3,830 (29.35)
4B	9,600	0.14	96.54	3,463 (36.08)
4D	2,700	0.03	218.80	1,71 (6.34)
5A	17,600	0.09	52.97	4,897 (27.83)
5B	22,750	0.14	40.22	8,193 (36.01)
5D	11,100	0.12	59.51	2,273 (20.48)
6A	14,700	0.08	57.51	3,928 (26.72)
6B	18,800	0.11	50.53	6,163 (32.78)
6D	7,950	0.05	87.75	911 (11.46)
7A	22,750	0.09	42.53	6,382 (28.05)
7B	22,450	0.11	42.47	7,492 (33.37)
7D	14,100	0.08	60.62	2,246 (15.93)
A genome	119,225	0.11	56.04	35,012 (29.44)
B genome	143,600	0.12	51.63	47,527 (33.24)
D genome	75,300	0.11	65.70	15,184 (20.16)
Total	338,125	0.11	57.79	97,723 (27.61)

*TNMP, total number of marker pairs; Mbp, mega-base pairs*.

### Genome-Wide Association Study

#### STB Resistance

The GWAS identified 53 SNPs that were significantly (FDR <0.05) associated with STB resistance at any growth stage. The report, however, only included the marker-trait associations (MTAs) that surpassed a Bonferroni-correction significance threshold of 0.15. Supplementary Table 3 presents the complete output of the GWAS results for STB resistance at the heading, mid-maturity, and maturity stages and for the Septoria progress coefficient. This table also reports allele identity, marker position, MAF, *p*-values, FDR-corrected q values, and the additive effects of the identified MTAs. The Q–Q plots demonstrating how well the used GWAS model accounted for population structure and kinship for STB resistance analysis are also presented in Supplementary Figure 2.

Among the 53 identified MTAs, 3 did not have chromosomal positions on the bread-wheat physical map (Supplementary Table 4). Ten (18.9%) of the MTAs conferred STB resistance at heading, 4 (7.6%) were effective at mid-maturity, 4 (7.6%) were effective at the maturity stage, and 35 (66.9%) of the MTAs were associated with a resistance to disease development as plant height increased (Supplementary Table 4). The percentage of phenotypic variance explained by the markers varied considerably, from 2.7% for the SDS measured at maturity at Bekojiin in 2016 to 13.2% for the severity data measured at the mid-maturity stage at the same location in 2015 (Supplementary Table 4). The proportion of phenotypic variation (*R*^2^) explained by SDS MTAs at heading ranged from 2.9% for the allele 1195254|F|0-31:C>T-31:C>T on chromosome 3A to 11.1% for 1087857|F|0-41:T>C-41:T>C on chromosome 7D (Supplementary Table 4). Likewise, the *R*^2^ for MTAs for SDS at mid-maturity, maturity, and SPC ranged from 8.6 to 13, 2.7 to 2.7, and 6 to 10.8%, respectively (Supplementary Table 4).

The combined measure of SDS at the heading, mid-maturity, and maturity stages did not provide any significant associations at the used threshold. However, the combined measure of SPC identified eight MTAs at the stringent Bonferroni significance threshold on chromosomes 1B, 2D, 3A, 3B, 3D, 6B, 7B, and 7D, with one MTA that was unmapped on the bread wheat physical map (Supplementary Table 4, Supplementary Figure 3). The GWA scan for SDS at the individual-environment level identified considerable (45) MTAs conferring resistance to STB at different growth stages (Supplementary Table 4, Supplementary Figures 4, 5). The analysis for disease data measured in 2015 at Holetta identified six MTAs for STB resistance at heading on chromosomes 1D, 2A, 3A, 3D, 5A, and 7D, four MTAs effective for STB resistance at the mid-maturity stage on chromosomes 1B, 3D, and 7B, with one MTA with an unknown position on the bread wheat physical map at Holetta, and nine MTAs for SPC (six at Holetta and three at Kulumsa) on chromosomes 1A, 1B, 1D, 2B, 3A, 3D, and 7B (Supplementary Table 4, Supplementary Figure 4). However, no MTA was observed for SDS data measured at the maturity stage in the same year. Likewise, the association analysis for SDS data measured in 2016 identified 26 MTAs: 4 for STB resistance at heading at Bekoji on chromosomes 3A, 3D, and 7A, with 1 MTA with an unknown position on the bread wheat physical map, 4 for maturity stage resistance on chromosomes 1D, 4A, 6A, and 7D at the same location, 18 MTAs for SPC, which were mapped to chromosomes 1A, 1B, 2B, 2D, 3B, 5B, 5D, 6A, 6B, 7A, and 7D plus 1 MTA with an unknown position on the bread wheat physical map (Supplementary Table 4, Supplementary Figure 5).

**Figure 5:**
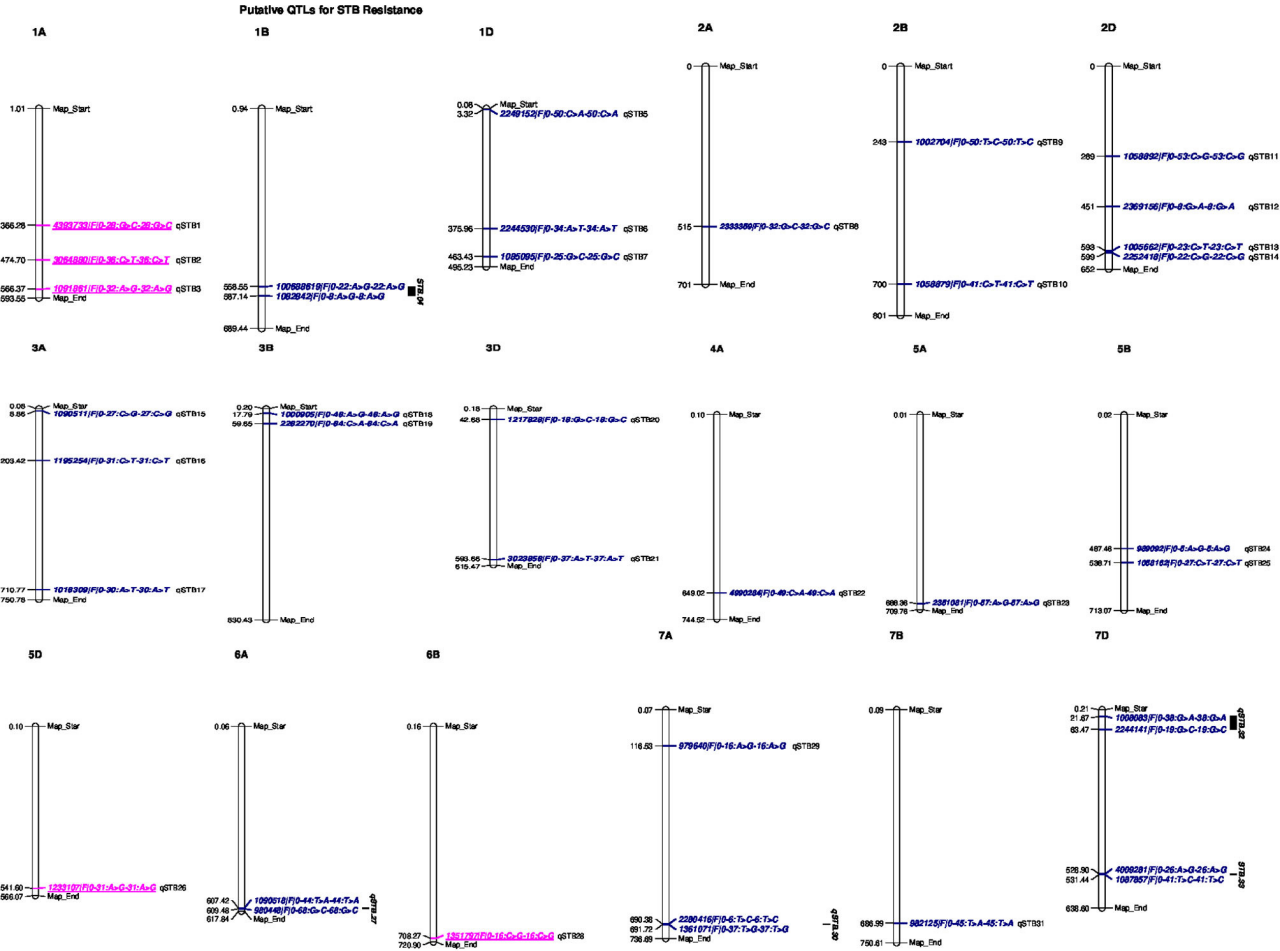
Genomic positions of detected putative QTLs effective for STB resistance. Significant DArTSeq SNPs are presented according to their physical positions on chromosomes in millions base pairs. The putative QTLs identified in this study for the MTAs are indicated on the right sides of the bars. Underlined MTAs (marked in pink) on the right sides of the chromosomes could be potentially novel loci in this study.

Putative QTL for STB resistance were identified by combining the MTAs based on their genomic positions using a window of physical distance (in Mbp) determined through a pair-wise LD analysis of the genome-wide scanned SNPs. Supplementary Figure 6 presents a scatter plot of the genome-wise pairwise LD r^2^ values between the SNPs on each chromosome against inter-marker physical distance. The MTAs falling on the same linkage group within the physical distance for LD decay specific for that chromosome were assigned to the same putative QTL. Hence, based on the LD criteria, the 53 markers were assigned to 33 putative QTLs (Table 8, Figure 5).

**Table 8:** Summary of the putative QTLs identified across bread wheat chromosomes for STB resistance.

**Putative QTL**	**Chr**	**Mapposition (bp)**	**No.of MTAs**		**Flanking Markers**		**Phenotype_Location_Year**	***R* ^**2**^**
				Position^*^ (Mbp)	**Left/Right**	Position^**^ (Mbp)		
qSTB.01	1A	366278319	1	366.28	9766808|F|0-10:A>G-10:A>G **/**1087379|F|0-64:G>A-64:G>A	374.57	SPC_Kulumsa_2016	6.91
qSTB.02	1A	474702375	1	474.56	4409931|F|0-10:T>C-10:T>C/2263809|F|0-17:G>C-17:G>C	475.71	SPC_Kulumsa_2015	9.21
qSTB.03	1A	566369413	1	565.20	987669|F|0-11:T>G-11:T>G/1863565|F|0-7:G>A-7:G>A	566.56	SPC_Kulumsa_2016	5.98
qSTB.04	1B	558551443-587138312	4	556.11	3948637|F|0-11:A>G-11:A>G/1276419|F|0-52:A>C-52:A>C	587.28	SPC and SDS at mid-maturity	5.98–9.67
qSTB.05	1D	3324483	1	2.95	2248863|F|0-53:G>T-53:G>T/1863120|F|0-61:T>C-61:T>C	4.82	SDSM_Bekoji_2016	273
qSTB.06	1D	375956648	1	363.25	1217216|F|0-11:G>C-11:G>C/1119123|F|0-11:C>G-11:C>G	377.58	SPC_Holetta_2015	9.66
qSTB.07	1D	463434850	1	462.07	1034027|F|0-63:C>G-63:C>G/1398976|F|0-52:G>C-52:G>C	464.86	SDSH_Holetta_2015	10.44
qSTB.08	2A	514858369	1	513.83	1181149|F|0-28:G>A-28:G>A/1102718|F|0-68:C>A-68:C>A	21.69	SDSH_Holetta_2015	10.63
qSTB.09	2B	243083729	1	237.98	100665389|F|0-10:A>G-10:A>G/3064852|F|0-14:G>A-14:G>A	249.19	SPC_Kulumsa_2016	6.63
qSTB.10	2B	700740191	1	698.10	1127049|F|0-20:T>C-20:T>C/1220715|F|0-13:A>G-13:A>G	701.09	SPC_Holetta_2015	9.84
qSTB.11	2D	288602370	1	215.60	3025921|F|0-19:G>T-19:G>T/1107980|F|0-6:T>C-6:T>C	331.55	SPC_Combined	9.68
qSTB.12	2D	450991087	1	443.24	2262159|F|0-55:C>A-55:C>A/991014|F|0-5:G>C-5:G>C	461.85	SPC_Bekoji_2016	7.35
qSTB.13	2D	593032041	1	591.60	2251911|F|0-13:A>G-13:A>G/1078056|F|0-40:C>T-40:C>T	594.54	SPC_Bekoji_2016	6.01
qSTB.14	2D	598728762	1	595.56	5324627|F|0-45:G>C-45:G>C/2246647|F|0-7:T>C-7:T>C	598.73	SPC_Kulumsa_2016	7.35
qSTB.15	3A	8862385	1	8.74	2256311|F|0-9:C>G-9:C>G/1088933|F|0-37:C>T-37:C>T	12.87	SPC_Combined	9.82
qSTB.16	3A	203418249	1	161.44	12470406|F|0-23:A>G-23:A>G/992022|F|0-9:G>A-9:G>A	222.04	SDSH_Bekoji_2016	2.92
qSTB.17	3A	710771071	2	710.34	989196|F|0-7:A>T-7:A>T/4989102|F|0-40:G>A-40:G>A	711.04	SDSH_Holetta_2015 SPC_Holetta_2015	9.92
qSTB.18	3B	17785833	1	17.11	1244651|F|0-19:A>G-19:A>G/998652|F|0-18:C>T-18:C>T	18.45	SPC_Kulumsa_2016	6.07
qSTB.19	3B	59645976	1	59.53	1263371|F|0-58:G>A-58:G>A/1110947|F|0-39:T>C-39:T>C	60.37	SPC_Combined	9.75
qSTB.20	3D	42679365	1	42.63	981546|F|0-39:T>C-39:T>C/4911094|F|0-6:T>C-6:T>C	45.94	SDSH_Bekoji_2016	3.48
qSTB.21	3D	593664469	5	593.66	1102020|F|0-37:G>A-37:G>A/992091|F|0-53:G>C-53:G>C	595.02	SDSH_Holetta_2015, SDSMM_Holeta_2015, SPC_Holetta_2015, SPC_Kulumsa_2015 and SPC_Combined	2.67–13.01
qSTB.22	4A	619375783	1	619.16	2263956|F|0-45:T>C-45:T>C/994022|F|0-52:G>C-52:G>C	620.75	SDSM_Bekoji_2016	2.7
qSTB.23	5A	688359748	1	685.96	3938163|F|0-43:T>C-43:T>C/2278701|F|0-37:A>T-37:A>T	689.42	SDSH_Holetta_2015	10.44
qSTB.24	5B	487460716	1	487.44	1696148|F|0-16:C>G-16:C>G/2281586|F|0-67:A>G-67:A>G	491.07	SPC_Kulumsa_2016	6.92
qSTB.25	5B	538706298	1	538.31	5582250|F|0-47:T>C-47:T>C/1097026|F|0-40:C>A-40:C>A	539.08	SPC_Bekoji_2016	6.92
qSTB.26	5D	541603929	1	539.15	1696148|F|0-16:C>G-16:C>G/6038202|F|0-6:C>T-6:C>T	541.68	SPC_Kulumsa_2016	6.13
qSTB.27	6A	607427728-609480220	2	607.43	2328288|F|0-13:G>C-13:G>C/1231806|F|0-13:G>C-13:G>C	608.28	SPC_Kulumsa_2016 and SPC_Kulumsa_2016	2.72–6.01
qSTB.28	6B	708272196	2	706.98	995556|F|0-65:C>T-65:C>T/1091698|F|0-30:C>G-30:C>G	708.02	SPC_Combined and SPC_Bekoji_2016	6.13–9.91
qSTB.29	7A	116530515	1	116.12	3064815|F|0-27:A>G-27:A>G/1151957|F|0-24:T>G-24:T>G	123.28	SPC_Kulumsa_2016	7.26
qSTB.30	7A	690377106-691722567	2	688.71	3064770|F|0-20:G>A-20:G>A/3532952|F|0-38:G>A-38:G>A	690.86	SPC_Bekoji_2016 and SDSH_Bekoji_2016	3.02–7.26
qSTB.31	7B	686989852	4	686.00	3023327|F|0-28:G>A-28:G>A/7340828|F|0-11:C>G-11:C>G	687.06	SDSMM_Holeta_2015, SPC_Holetta_2015, SPC_Kulumsa_2015 and SPC_Combined	8.71–9.71
qSTB.32	7D	21667638-63471279	3	18.92	1072451|F|0-9:C>T-9:C>T/981671|F|0-20:G>C-20:G>C	64.24	SDSM_Bekoji_2016, SPC_Combined and SPC_Bekoji_2016	2.67–10.79
qSTB.33	7D	528900507-531439751	2	526.49	1004225|F|0-30:G>A-30:G>A/5579572|F|0-19:G>A-19:G>A	540.62	SPC_Bekoji_2016 and SDSH_Holetta_2015	6.63–11.09

QTL, quantitative trait locus; Chr, chromosome; MTAs, marker-trait associations; Position^*^, position (Mbp) of the left flanking marker; Position^**^, position (Mbp) of the right flanking marker; Phenotype_Location_year, Septoria disease severity measured at individual or across location levels (Holetta, Bekoji, and Kulumsa) in 2015 and 2016; R^2^, percentage of the total phenotypic variance explained by the identified putative QTL; SDSH, Septoria disease severity at heading; SDSMM, Septoria disease severity at mid-maturity; SDSM, Septoria disease severity at maturity; SPC, Septoria progress coefficient.

The association analysis for STB resistance at heading in the individual environments identified nine putative QTL localized on chromosomes 1D (qSTB.07), 2A (qSTB.08), 3A (qSTB.16 and qSTB.17), 3D (qSTB.20 and qSTB.21), 5A (qSTB.23), 7A (qSTB.30), and 7D (qSTB.33) (Table 8, Figure 5). The combined measure of the SDS at the mid-maturity stage did not reveal any QTL. However, measuring the same trait in 2015 at Holetta identified three putative QTLs on chromosomes 1B (qSTB.04), 3D (qSTB.21), and 7B (qSTB.31) (Table 8) that were effective for STB resistance at the mid-maturity stage. Likewise, the SDS measured at maturity in 2016 at Bekoji provided four putative QTLs on chromosomes 1D (qSTB.05), 4A (qSTB.22), 6A (qSTB.27), and 7D (qSTB.32) (Table 8, Figure 5). However, the same phenotype measured across all environments did not provide any putative QTLs effective for STB resistance.

We identified seven QTLs for the SPC in the analysis of means over all the six environments: qSTB11 on 2D; qSTB15 on 3A; qSTB19 on 3B; qSTB21 on 3D; qSTB28 on 6B; qSTB31 on 7B; qSTB32 on 7D. These seven QTL modeled 9.7 to 13% of the phenotypic variation. Three of these QTLs (qSTB.11 on 2D, qSTB.14 on 3A, and qSTB.19 on 3D) were not significant in the analyses of the six environments; two (qSTB.28 and qSTB.32) were significant in one of the environments; the other two (qSTB.21 and qSTB.31) were significant in two environments (Table 8, Figure 5). Measuring the same trait in 2015 identified seven putative QTLs on chromosomes 1A (qSTB.01), 1B (qSTB.04), 1D (qSTB.06), 2B (qSTB.10), 3A (qSTB.17), 3D (qSTB.21), and 7B (qSTB.31) (Table 8, Figure 5). Similarly, the association analysis for the SPC data measured during 2016 identified effective putative QTLs on chromosomes 1A (qSTB.01 and qSTB.03), 1B (qSTB.04), 2B (qSTB.09), 2D (qSTB.12-14), 3B (qSTB.18), 5B (qSTB.24 and qSTB.25), 5D (qSTB.26), 6A (qSTB.27), 6B (qSTB.28), 7A (qSTB.29), and 7D (qSTB.32 and qSTB.33) (Table 8, Figure 5).

The functional association of the identified QTLs for STB resistance was further investigated by annotating genes found in the QTL regions on the recently released IWGSC RefSeq Annotation v2. The analysis resulted in several disease resistance-associated genes involved in plant defense systems (Supplementary Table 7). For instance, the high-confidence candidate genes *TraesCS1A02G279300* on 1A, *TraesCS1B02G332400* on 1B, *TraesCS1D02G001700* on 1D, and *TraesCS2A02G297500* on 2A are highly involved in systemic acquired resistance (SAR), which refers to the long-lasting, broad-spectrum resistance of plants to pathogen infections. Specifically, the high-confidence gene detected near *qSTB.08* on chromosome 2A (*TraesCS2A02G297500*) controls mitogen-activated protein kinase (MAPK) cascades, which are involved in signaling multiple defense responses of plants against pathogen attacks (Meng and Zhang, 2013).

### MTAs for Agronomic Traits

The combined measures of the agronomic traits, such as days to heading, days to flowering, days to maturity, grain-filling duration, grain yield, and 1,000-kernel weight, did not provide any MTAs at the stringent Bonferroni significance threshold used except for plant height, which resulted in one MTA on chromosome 7A at the 514.43 Mbp position (Supplementary Table 6). However, the dissections of these traits in the individual environments in separate years identified considerable MTAs at the significance threshold utilized (Supplementary Figure 7). In particular, a GWA scan for days to heading in 2015 at Holetta provided six MTAs on chromosomes 1A, 5A, 5B, 6A, 6B, and 7B (Supplementary Table 6). The same trait measured in 2016 at Kulumsa identified three significant (FDR <0.05) SNPs on chromosomes 2B, 5D, and 6A (Supplementary Table 6). Additionally, the total phenotypic variance explained by the SNPs for this trait ranged from 8.5% for the allele 987806|F|0-16:A>G-16:A>G on chromosome 5D at 77.02 Mbp to 24.1% for the SNP1204551|F|0-57:C>T-57:C>T positioned on chromosome 1A at 499.84 Mbp (Supplementary Table 6).

The GWA scan for days to flowering in the individual environments identified six MTAs for the data measured in 2015 at Holetta on chromosomes 1A, 5A, 5B, 6A, 6B, and 7B (Supplementary Table 6, Supplementary Figure 8). The detected markers could explain 22.8–25% of the total phenotypic variation for days to flowering, while the SNP markers 1000134|F|0-15:T>C-15:T>C on chromosome 6B and 1204551|F|0-57:C>T-57:C>T on chromosome 1A accounted for the lowest and highest phenotypic variations in days to flowering in the association panel, respectively (Supplementary Table 6). Likewise, the association analysis for the days to maturity data measured in 2016 at Bekoji identified 15 MTAs pointing to nine putative QTLs on 1A, 2A, 3A, 3B, 5B, 6D, and 7A (Supplementary Table 6, Supplementary Figure 9). Three of these significant SNPs, however, were not mapped on the bread wheat physical map. The detected markers explained 11.6–15.6% of the total phenotypic variation for days to maturity, while the lowest and highest values were reported for the SNPs 2278215|F|0-18:A>G-18:A>G on 2A and for 7332831|F|0-9:T>C-9:T>C on 1A, respectively (Supplementary Table 6).

Moreover, a GWA scan on grain-filling duration data measured in 2016 revealed 15 MTAs, among which 11 were identified from the data collected at Holetta on chromosomes 1B, 2B, 3A, 3B, 6D, and 7D, plus 1 MTA with an unknown position on the bread wheat physical map (Supplementary Figure 9). Four of the significant associations for this trait were obtained from data measured at Kulumsa on chromosomes 2B, 3B, 5B, and one MTA with an unknown chromosomal position on the bread wheat genome. The total phenotypic variation for GFD explained by the SNPs ranged from.5% for the allele on chromosome 3B (5325155|F|0-26:G>T-26:G>T) to 7.9% for the SNP marker on 3A at 250.82 Mbp (5325155|F|0-26:G>T-26:G>T) (Supplementary Table 6).

The association analysis for pooled plant height data identified 1 MTA on chromosome 7A (Supplementary Figure 9) and 24 for the data measured in the individual environments (Supplementary Table 6). The plant height data measured in 2015 at Bekoji resulted in 4 MTAs on chromosomes 1A, 5A,7A, and 7B and 10 MTAs for Kulumsa on chromosomes 1B, 2A, 5B, 5D, 6B, and 7D (Supplementary Figures 7, 8). The same trait measured in 2016 at Kulumsa provided 10 MTAs on chromosomes 3B, 5A, 5B, 6B, 6D, and 7D, with 2 MTAs that were unmapped on the bread wheat physical map (Supplementary Table 6, Supplementary Figure 9). The identified SNPs accounted for 3.3–12.3% of the total variation in plant height, and the SNP marker on 6B (SNP 2276919|F|0-10:G>T-10:G>T) at 521.99 Mbp had the largest effect (Supplementary Table 6).

The study revealed that grain yield and yield-related attributes measured over all the environments did not provide any significant SNPs. However, their association analysis based on mean values in the individual environments identified numerous MTAs. The grain yield measured in 2015 at Kulumsa identified 10 significant MTAs on chromosomes 1A, 3A, 3D, 4A, 5A, 5D, and 7D, with one SNP that was not mapped in the bread wheat genome (Supplementary Table 6, Supplementary Figure 8). Similarly, a GWA scan for TKW measured in 2016 at Kulumsa provided one MTA pointing to a putative QTL on 7D at 79.52 Mbp (Supplementary Table 6, Supplementary Figure 9). Furthermore, the total phenotypic variations explained by the significant SNPs for yield and yield-related traits varied considerably based on traits and markers, with the lowest being 0.5% for grain yield on 3D (1045011|F|0-60:T>A-60:T>A) and the highest being 15.7% for TKW on 7D (4262368|F|0-28:A>G-28:A>G) (Supplementary Table 6).

The analysis revealed that some putative QTLs identified by SDS data overlapped with those determined for agronomic data. For instance, the putative QTL determined for plant height measured in 2016 at Kulumsa on chromosome 6B was co-mapped with qSTB.28, which was identified for combined SPC. Similarly, the putative QTL identified on 7B for plant height measured at Bekoji in 2015 was co-mapped with qSTB.31, which was also identified for combined SPC. In addition, the putative QTLs mapped on chromosome 7D for plant height measured in 2015 and 2016 at Kulumsa and for GFD and TKW data measured in 2016 at Holetta and Kulumsa, respectively, were co-mapped with qSTB.32, which was identified for the SDS data measured at the maturity stage at Bekoji and for pooled SPC data.

## Discussion

The phenotypic evaluation revealed significant genetic variability for STB resistance among the tested wheat genotypes, thus confirming the availability of relevant alleles for future breeding and improvement. The observed high broad-sense heritabilities within the individual environments (H^2^ = 0.58–0.99) and across the environments (H^2^ = 0.72–81) indicated a strong genetic signal in the data, which can be used for improving STB resistance through selection. Similarly, a high broad-sense heritability (H^2^ = 0.78) for STB resistance was reported for European bread wheat varieties in Germany (Muqaddasi et al., 2019) and Tunisia (H^2^ = 0.55) (Berraies et al., 2014). The present field evaluation results confirmed that STB infestation is significantly influenced by year, location, and all interaction effects, thus confirming the importance of multiple locations and years for germplasm evaluations at disease hotspots to identify durable and stable STB-resistant genotypes.

The correlation analysis revealed that Septoria disease ratings had negligible negative correlations with plant height, indicating that tallness only had a weak contribution for reducing STB infections (Muqaddasi et al., 2019). The lack of or slight negative correlations of SDS traits with the agronomic traits HD, FD, GFD, NKPS, and NKS and the moderately negative correlation with MD indicate that genotypes with late phenology could escape STB with slightly reduced infection. Moreover, the significant negative correlations of STB infection with yield and yield-related traits such as HLW, TKW, and KN could most likely be due to the fact that the infection of the flag and second leaves at the grain-filing stage could significantly influence the photosynthesis process, and, thus, result in reduced grain yield. Negative associations of SDS with days to flowering, days to maturity, number of seeds per spike, and thousand-grain weights were also reported for Ethiopian durum wheat (Kidane et al., 2017).

The STRUCTURE and principal component analyses revealed population stratifications and admixtures, suggesting the need to use a powerful statistical model in the association analysis that controls for spurious marker-trait associations. The analyses suggested two sub-groups in the population. The very powerful statistical model used in the analysis, FarmCPU, sufficiently accounted for population stratification, familial relatedness, and marker effects, which consequently reduced the confounding effects that could result in false-positive MTAs. This was confirmed by visualizing the Q–Q and Manhattan plots. Similar indistinct population stratifications, higher admixtures, and weak population sub-structuring were reported among 371 European wheat genotypes based on 35k and 90k SNP marker arrays (Muqaddasi et al., 2019).

Like previous reports, this study confirmed the unequal distribution of the SNP markers among wheat genomes, where most were harbored by the A (10,317) and B genomes (10,979), while fewer SNPs (9,756) were harbored by the D genome (Berkman et al., 2013; Edae et al., 2015; Rahimi et al., 2019). This variation most likely resulted from the evolutionary and domestication history of the crop (Dvorak et al., 2006; Jordan et al., 2015), where the D genome had less time to accumulate mutations.

These analyses revealed that the LD between the markers and genes contributing to STB resistance declined to *r*^2^ <0 within a physical distance of 1.26–105.61 Mbp in all the chromosomes, with an overall mean of 31.44 Mbp. This is much lower than the average physical distance (69.1 Mbp) for LD decay in Ethiopian durum wheat at the critical threshold of *r*^2^ = 0.2 (Alemu et al., 2021). The marker distances at which the LD decayed across the older sub-genomes (A and B) were relatively lower than those for the D sub-genome, most likely because of the long evolutionary history of the A and B genomes as compared with the D genome. Furthermore, the LD between alleles can decay because of a number of factors such as selection, recombination, the mating system, genetic drift, mutation, and/or population relatedness (Stich and Melchinger, 2010). Hence, it is likely that the shorter selection history of the D sub-genome did not allow linkage breakdown due to the recombination that occurs between SNPs located at longer physical distances.

The GWAS analysis identified 53 MTAs pointing to 33 QTL for STB resistance and 82 MTAs for agronomic traits where markers had a FDR *p* ≤ 0.05 and a Bonferroni correction significance threshold of 0.15. The number of SDS MTAs identified in this study was significantly lower than that in the findings of Rahimi et al. (2019), who reported 313–394 MTAs for an Iranian bread wheat association panel. However, this number was still substantially higher than that in the report of Kidane et al. (2017), who identified 35 significant associations for an Ethiopian durum wheat panel. Only seven QTLs for SPC were identified in an analysis of the mean over environments, while none was detected for SDS. Kidane et al. (2017) also reported no QTLs for SDS in an analysis of means.

More QTL were noted in the analysis of data from individual environments, although none was detected in more than four of the six environments and 24 of the 33 were detected in just one environment. The failure to detect QTL effects over environments could be due to the seasonal specificity of QTL effects, disease pressure, or the use of a very stringent FDR level that controls type I errors but leads to increased type II errors, e.g., declaring a QTL not significant when it actually is. The 2015 growing season at Holetta was characterized by extended and heavy rainfall that resulted in the highest STB natural infestations across all the growth stages. Additionally, in this growing season, 12 individual QTLs were detected (five for SPC and seven for SDS), 3 of which affected both SPC and SDS. In contrast, the heavy rainfall and prolonged moisture experienced at Bekoji in 2016 produced the highest SDS, while 12 QTLs were detected, of which 6 were for SPC, 4 for SDS, and 2 that affected both traits, using the data that were obtained. Therefore, climatic conditions, such as persistent crop moisture and prolonged heavy rain, favor the successful infection and spread of the disease throughout the crop canopy (Fones and Gurr, 2015). Furthermore, no QTLs for SDS were detected for Kulumsa in either year. Although a total of 11 QTLs for SPC were detected in the same environment, none was repeated over the years. The different climatic conditions may have caused the later onset of the disease in both growing seasons. However, one QTL, qSTB.21, had the most repeatable effect and was significant for SPC overall for both SPC in two environments SDS at the heading and mid-maturity stages in 2015 at Holetta.

In this study, the GWA scan on SDS data measured at heading at Holetta in 2015 and at Bekoji in 2016 identified putative QTLs on chromosomes 1D, 2A, 3A, 3D, 5A, 7A, and 7D. Moreover, the association analysis for SDS at the mid-maturity stage at Holetta in 2015 reported three effective putative QTLs on chromosomes 1B, 3D, and 7B, which have not been reported for this trait so far. Likewise, dissecting the disease data measured at the maturity stage identified putative QTLs on chromosomes 1D, 4A, 6A, and 7D. Moreover, the GWA scan for SPC identified putative QTLs on chromosomes 1A, 1B, 1D, 2B, 2D, 3A, 3B, 3D, 5B, 5D, 6A, 6B,7A, 7B, and 7D.

Although the different mapping methods, marker systems, and populations used can make it difficult to compare QTL positions from different studies, some of the QTLs detected in this analysis coincided with the mapping positions of previously reported major STB resistance genes. Hence, the putative QTL on 1B (qSTB.04) may represent *Stb2* (Liu et al., 2013) and/or *Stb11* (Chartrain et al., 2009), the QTLs on 1D (qSTB.05–07) may represent *Stb10* (Chartrain et al., 2005), the QTL on 2B may represent *Stb9* (Chartrain et al., 2009), the QTLs on 3A (qSTB.15–17) may represent *Stb6* (Brading et al., 2002) and/or *StbSm3* (Cuthbert, 2011), the QTLs on 3B may represent *Stb14* (Cowling, 2006), the QTLs on 3D may represent *Stb16q* (Tabib Ghaffary et al., 2012), the QTL on 4A may represent *Stb7* (McCartney et al., 2003) or *Stb12* (Chartrain et al., 2005), the QTLs on 5A may represent *Stb17* (Tabib Ghaffary et al., 2012), the QTLs on 5B may represent *Stb1* (Adhikari et al., 2004a), the QTL on 6A may represent *Stb15* (Arraiano et al., 2007), the QTLs on 7A may represent *Stb3* (Goodwin and Thompson, 2011) or *TmStb1* (Jing et al., 2008), the QTL on 7B may represent *Stb8* (Adhikari et al., 2003) or *Stb13* (Cowling, 2006), and the putative QTLs on 7D may represent *Stb4* (Adhikari et al., 2004b) or *Stb5* (Arraiano et al., 2001). Similar to the results of this study, Kollers et al. (2013) also reported significant MTAs for STB resistance on chromosomes 2A and 2D. The present result also agrees with Muqaddasi et al. (2019), who reported an adult-plant-stage STB resistance QTL on chromosome 4A.

The identification of defense-related candidate genes, such as *TraesCS1A02G279300, TraesCS1B02G332400, TraesCS1D02G278400, TraesCS2B02G233600, TraesCS2A02G297500, TraesCS2D02G497400, TraesCS2D02G506300*, and *TraesCS4A02G341300*, in the vicinity of the significant markers indicates the possible functional association of the detected QTL regions in plant defense systems against pathogen infections. For instance, the translations of the genes found in qSTB.02 (*TraesCS1A02G279300*) and qSTB.04 (*TraesCS1B02G332400*) on chromosomes 1A and 1B, respectively, are involved in the jasmonic- acid and ethylene-dependent systemic-acquired resistance of plants to pathogen infections. This systemic acquired resistance (SAR) is a broad-spectrum, long-lasting resistance acquired after the initial localized infection of plants by pathogens (Lawton et al., 1995). Furthermore, the gene *TraesCS2A02G297500* found in qSTB.08 on chromosome 2A controls MAPK cascades, which are involved in signaling multiple defense responses, including the biosynthesis/signaling of plant stress/defense hormones, reactive oxygen species (ROS) generation, stomatal closure, defense gene activation, phytoalexin biosynthesis, cell wall strengthening, and hypersensitive response (HR) cell death. Moreover, most of the genes in proximity to the detected significant markers are inferred to be involved in salicylic acid (SA) biosynthesis, with SA being an important plant hormone that is best known for mediating host responses upon pathogen infection (Lefevere et al., 2020).

In this study, we discovered some STB resistance QTL that appear to be novel. These include the putative QTLs on chromosomes 1A (qSTB.01-3), 5D (qSTB.26), and 6B (qSTB.28) that explained >5% of the genetic variations, suggesting their relevance for wheat resistance breeding against STB. To the best knowledge of the authors, none of the known major STB resistance genes published in existing literature have been mapped to these regions of the wheat chromosomes; therefore, these QTLs could be considered novel.

Moreover, the study revealed that some of the putative STB resistance QTLs were co-located with QTL for agronomic traits. For instance, the putative QTLs derived from plant height measured in 2016 at Kulumsa on chromosome 6B (*R*^2^ = 11.36) and in 2015 at Bekoji on chromosome 7B (*R*^2^ = 8.88) were co-mapped with qSTB.28 and qSTB.31, which were identified for combined SPC. Likewise, the putative QTLs mapped on chromosome 7D for grain-filling duration (*R*^2^ = 4.69) and 1,000-kernel weight measured in 2016 (*R*^2^ = 0.54) at Holetta and Kulumsa, respectively, were co-mapped with qSTB.32, which was identified for the SDS data measured at the maturity stage at Bekoji and for the pooled SPC data. Furthermore, STB is the most destructive foliar disease in Ethiopia. Hence, the infection of the flag and second leaves, which contributes most to photosynthetic assimilates at the grain-filling stage (King et al., 1983; Muqaddasi et al., 2019), can result in the substantial loss of grain weight and yield. This is consistent with the findings of Kidane et al. (2017), who reported the co-mapping of putative QTLs for 1,000-kernel weight with SDS data. Moreover, the putative QTL on chromosome 1A identified for grain yield measured at Kulumsa in 2015 (*R*^2^ = 0.79) was co-mapped with qSTB.03, which was identified for the SPC measured in the same environment. It is, therefore, expected that the vertical progression rate of the disease could affect grain yield by influencing grain filling and the number of seeds produced per spike.

In this study, most of the QTLs identified for agronomic and phenological traits did not overlap with those detected for SDS traits, likely because of the lack of common genetic effects for STB resistance and these traits. Many of the correlations of STB traits with agronomic traits were non-significant and had negligible to weak negative coefficients, indicating that the traits were independent. However, some level of co-localization was observed for the putative QTL for days to heading and days to flowering measured at Holetta in 2015 on chromosome 6B (*R*^2^ = 22.82), with the putative QTL qSTB.28 on 6B being identified for the SPC measured at Bekoji in 2016 and for the pooled data.

## Conclusions

In this study, the genetic architecture of adult-plant resistance to STB was explored in bread wheat using high-density, genome-wide SNP markers and multi environment-derived phenotype data. The analysis revealed that the association panel possessed considerable STB resistance alleles that could be deployed to improve wheat resistance to the prevailing *Z. tritici* populations in Ethiopia. Several genotypes with better resistance than the moderately resistant check King-bird were identified. Furthermore, the GWAS identified 33 putative QTLs, which were associated with 53 SNPs. Most (24) of the QTLs were detected in just one environment, suggesting the presence of resistance gene/genes effective against location-specific *Z. tritici* races. The detected QTLs also explained 2.7–13.2% of the total phenotypic variance for STB resistance. Several disease resistance-associated gene/s were also identified in the proximity of the detected SNPs, which can be targeted in efforts to understand the actual causative genes at the associated loci. Additionally, most of the detected putative QTLs shared similar chromosomal positions with previously reported genes and QTLs. Among these detected alleles, five were potentially novel, accounting for >5% of STB resistance. However, the effects of these QTLs need to be validated before being deployed in MAS. Finally, we conclude that the identified stably resistant wheat genotypes and the identified QTLs can be deployed in wheat breeding programs for the development of durable and broad-spectrum-resistant varieties against *Z. tritici*.

## Data Availability Statement

The original contributions presented in the study are publicly available. This data can be found here: Dryad data repository doi: 10.5061/dryad.7sqv9s4s4.

## Author's Note

We confirm that the manuscript has been read and approved by all the named authors and that there are no other persons who satisfied the criteria for authorship but are not listed. We further confirm that the order of authors listed in the manuscript has been approved by all of us.

## Author Contributions

TM was involved in the conceptualization, methodology, statistical analysis, and writing of the original draft. CS was involved in genotyping and editing. TH was involved in data curation and visualization. BA contributed material, performed the field experiments, and edited. CZ was involved in investigation and validation. DL was involved in reviewing. SG was involved in editing. KT was involved in methodology, funding acquisition, project administration, and supervision. All the authors approved the final version of the manuscript.

## Funding

This study was supported financially by the Ministry of Innovation and Technology (formerly Ministry of Science and Technology) of the Federal Democratic Republic of Ethiopia.

## Conflict of Interest

The authors declare that the research was conducted in the absence of any commercial or financial relationships that could be construed as a potential conflict of interest.

## Publisher's Note

All claims expressed in this article are solely those of the authors and do not necessarily represent those of their affiliated organizations, or those of the publisher, the editors and the reviewers. Any product that may be evaluated in this article, or claim that may be made by its manufacturer, is not guaranteed or endorsed by the publisher.

## Abbreviations

DH: days to heading
DF: days to flowering
DM: says to maturity
FDR: false discovery rate
FarmCPU: fixed and random model circulating probability unification
GAPIT: genome association and prediction integrated tools
GBS: genotyping by sequencing
GFD: grain-filling duration
GWAS: genome-wide association study
HLW: hectoliter weight
PH: plant height
LD: linkage disequilibrium
MAF: minor allele frequency
MAS: marker-assisted selection
MTAs: marker-trait associations
PCA: principal component analysis
QTL: quantitative trait locus or loci
SL: spike length
SN: seed number per spike
SDS: Septoria disease severity
SDSH: Septoria disease severity at heading
SDSMM: Septoria disease severity at mid-maturity
SDSM: Septoria disease severity at maturity
STB: Septoria tritici blotch
SNP: single-nucleotide polymorphism
SPC: Septoria progress coefficient
TKW: thousand kernel weight.
