# Supplementary material for: Genome-Wide Association Study Reveals Novel Genetic Loci for Quantitative Resistance to Septoria Tritici Blotch in Wheat (Triticum aestivum L.)
Source: Front Plant Sci. 2021 Sep 24;12:671323. doi: 10.3389/fpls.2021.671323 (PMC8500178; doi:10.3389/fpls.2021.671323)
Supplement: Supplementary file 7 [file Table_7.docx]

Supplementary Table 7. High confidence genes and gene models detected within the identified STB resistance associated QTL regions.

| Putative QTL | Chromosome | SNP Position | Gene ID ^a^ | | | | Gene/gene model position Gene names | | | | | | | | Gene Ontology (Biological process) |
| --- | --- | --- | --- | --- | --- | --- | --- | --- | --- | --- | --- | --- | --- | --- | --- |
|  |  |  |  |  |  |  | Start | | | End | | | strand | |  |
| qSTB.01 | 1A | 366278319 | TraesCS1A02G201700 | | | | 363006018 | | | 363007340 | | | - | | Defense response/ethylene biosynthetic process. |
|  |  |  | TraesCS1A02G203900 | | | | 365617809 | | | 365619048 | | | - | | Indoleacetic acid biosynthetic process/Regulation of defense response |
|  |  |  | TraesCS1A02G202000 | | | | 363047773 | | | 363052288 | | | - | | Immune system process. |
| qSTB.02 | 1A | 474702375 | TraesCS1A02G279300 | | | | 475309490 | | | 475313192 | | | + | | Systemic acquired resistance/ jasmonic acid mediated signaling pathway/regulation of plant-type hypersensitive/ defense response to fungus/defense response to bacterium |
| qSTB.03 | 1A | 566369413 | TraesCS1A02G403900 | | | | 567318284 | | | 567327266 | | | + | | cellular response to stimulus, Hessian fly resistance |
| qSTB.04 | 1B | 558551443-587138312 | TraesCS1B02G333500 | | | | 560018875 | | | 560021191 | | | - | | Response to biotic stress |
|  |  |  | TraesCS1B02G332400 | | | | 558550247 | | | 558552802 | | | - | | Jasmonic acid metabolic process/jasmonic acid and ethylene-dependent systemic resistance/induced systemic resistance |
| qSTB.05 | 1D | 3324483 | TraesCS1D02G001700 | | | | 447129 | | | 462647 | | | + | | Systemic acquired resistance/regulation of defense response. |
| qSTB.06 | 1D | 375956648 | TraesCS1D02G276100 | | | | 372147878 | | | 372155789 | | | + | | defense response |
|  |  |  | TraesCS1D02G278400 | | | | 375954117 | | | 375957575 | | | - | | Jasmonic acid mediated signalling pathway/ethylene-activated signaling pathway. |
| qSTB.07 | 1D | 463434850 | TraesCS1D02G389000 | | | | 460147069 | | | 460151558 | | | - | | Response to biotic stress |
|  |  |  | TraesCS1D02G387600 | | | | 460147069 | | | 460151558 | | | - | | Defense response to biotic stresses |
|  |  |  | TraesCS1D02G393000 | | | | 462739002 | | | 462746635 | | | - | | Systemic acquired resistance/regulation of plant-type hypersensitive response /defense response to fungus. |
|  |  |  | TraesCS1D02G390300 | | | | 461536153 | | | 461539106 | | | - | | Response to biotic stress |
|  |  |  | TraesCS1D02G390100 | | | | 461536153 | | | 461539106 | | | - | | Response to abiotic stress, response to biotic stress |
|  |  |  | TraesCS1D02G387600 | | | | 460147069 | | | 460151558 | | | - | | Defense response. |
| qSTB.08 | 2A | 514858369 | TraesCS2A02G295900 | | | | 509070654 | | | 509076530 | | | + | | Response to biotic stress |
|  |  |  | TraesCS2A02G299900 | | | | 515452029 | | | 515453761 | | | + | | Programmed cell death/ induction of programmed cell death. |
|  |  |  | TraesCS2A02G298600 | | | | 514231854 | | | 514233503 | | | + | | Defense response to fungus. |
|  |  |  | TraesCS2A02G298100 | | | | 513286315 | | | 513287713 | | | + | | Defense response. |
|  |  |  | TraesCS2A02G297500 | | | | 511474142 | | | 511477203 | | | + | | Defense response to fungus, plant-type hypersensitive response, systemic acquired resistance/ salicylic acid mediated signaling pathwa/ jasmonic acid mediated signaling pathway/ **MAPK cascades** are involved in signaling multiple **defense** responses, including the biosynthesis/signaling of **plant** stress/ **defense** hormones, reactive oxygen species (ROS) generation, stomatal closure, **defense** gene activation, phytoalexin biosynthesis, cell wall strengthening, and hypersensitive response (HR) cell death. |
| qSTB.09 | 2B | 243083729 | TraesCS2B02G233600 | | | | 232266510 | | | 232273397 | | | + | | Systemic acquired resistance/Jasmonic acid biosynthetic process/ resistance response to pathogenic bacteria |
|  |  |  | TraesCS2B02G492500 | | | | 690682191 | | | 690683084 | | | - | | Obsolete pathogen-associated molecular pattern dependent induction by symbiont of host innate immune response |
|  |  |  | TraesCS2B02G500100 | | | | 695688695 | | | 695689617 | | | + | | Abscisic acid-activated signaling pathway/response to biotic stimulus/defense response. |
|  |  |  | TraesCS2B02G499300 | | | 695449352 | | | | 695451719 | | | + | | Response to stress |
| qSTB.11 | 2D | 288602370 | TraesCS2D02G246800 | | | 289157650 | | | | 289160782 | | | + | | Response to biotic stress |
| qSTB.12 | 2D | 450991087 | TraesCS2D02G350500 | | | 448501026 | | | | 448502188 | | |  | | Response to biotic stress |
|  |  |  | TraesCS2D02G348700 | | | 446640621 | | | | 446640884 | | | - | | Response to biotic stress |
|  |  |  | TraesCS2D02G351600 | | |  | | | |  | | |  | | Jasmonic acid mediated signaling pathway. |
| qSTB.13 | 2D | 593032041 | TraesCS2D02G489700 | | | 588676152 | | | | 588677693 | | | - | | Response to biotic stress |
|  |  |  | TraesCS2D02G486800 | | | 586957083 | | | | 586961710 | | | - | | Defense response to fungus. |
|  |  |  | TraesCS2D02G488100 | | | 587696143 | | | | 587696631 | | | + | | Response to biotic stress |
|  |  |  | TraesCS2D02G488200 | | | 587697352 | | | | 587701032 | | | - | | Response to biotic stress |
|  |  |  | TraesCS2D02G497700 | | | 593408267 | | | | 593410141 | | | + | | Response to biotic stress |
|  |  |  | TraesCS2D02G497600 | | | 593370558 | | | | 593373483 | | | + | | Response to biotic stress |
|  |  |  | TraesCS2D02G497400 | | | 593270400 | | | | 593275755 | | | - | | Salicylic acid mediated signaling pathway/defense response to fungus/abscisic acid-activated signaling pathway/ jasmonic acid mediated signaling pathway. |
|  |  |  | TraesCS2D02G498000 | 593523002 | | | | | | 593524197 | | | - | | Methyl jasmonate esterase activity |
| qSTB.14 | 2D | 598728762 | TraesCS2D02G504500 | 598812395 | | | | | | 598819350 | | | + | | Biotic defense response. |
|  |  |  | TraesCS2D02G504200 | 598733638 | | | | | | 598735164 | | | _ | | Salicylic acid-dependent systemic acquired resistance. |
|  |  |  | TraesCS2D02G504000 | 598714158 | | | | | | 598720273 | | | - | | Response to biotic stress |
|  |  |  | TraesCS2D02G503900 | 598712612 | | | | | | 598713618 | | | + | | Response to biotic stress |
|  |  |  | TraesCS2D02G503800 | 598610615 | | | | | | 598613095 | | | + | | Defense response: defense/immunity protein activity |
|  |  |  | TraesCS2D02G506300 | 600161338 | | | | | | 600164249 | | | - | | Salicylic acid mediated response to a pathogen which confers broad spectrum resistance/salicylic acid-dependent systemic resistance |
|  |  |  | TraesCS2D02G506200 | 600131157 | | | | | | 600134165 | | | - | | Salicylic acid mediated response to a pathogen which confers broad spectrum resistance |
|  |  |  | TraesCS2D02G505700 | 599916155. | | | | | |  | 599919148 | + | | Jasmonic acid biosynthetic process. | |
| qSTB.15 | 3A | 8862385 | TraesCS3A02G007800 | 7788947 | | | | | | 7791684 | | | - | | Response to biotic stress |
|  |  |  | TraesCS3A02G007000 | 7432258 | | | | | | 7432761 | | | - | | Response to biotic stress |
|  |  |  | TraesCS3A02G005500 | 6891942 | | | | | | 6899514 | | | - | | Immune system process: defense response |
|  |  |  | TraesCS3A02G016500 | 7469796 | | | | | | 7474094 | | | - | | Response to biotic stress |
|  |  |  | TraesCS3A02G010900 | 9064431 | | | | | | 9066825 | | | - | | Defense response to fungus |
|  |  |  | TraesCS3A02G009900 | 8837590 | | | | | | 8840754 | | | + | | Defense response |
|  |  |  | TraesCS3A02G009800 | 8794573 | | | | | | 8800596 | | | - | | Defense response to fungus |
|  |  |  | TraesCS3A02G009300 | 8549930 | | | | | | 8550907 | | | + | | Defense response. |
|  |  |  | TraesCS3A02G009200 | 8323975 | | | | | | 8330044 | | - | | | Defense response |
|  |  |  | TraesCS3A02G019400 | 11800250 | | | | | | 11808293 | | - | | | Response to biotic stress |
|  |  |  | TraesCS3A02G020700 | 11808293 | | | | | | 11808293 | | - | | | Salicylic acid mediated signaling pathwa |
| qSTB.16 | 3A | 203418249 | TraesCS3A02G479600 | 711077944 | | | | | | 711078848 | | - | | | Response to biotic stress. |
| qSTB.17 | 3A | 710771071 | TraesCS3A02G479400 | 711056566 | | | | | | 711061191 | | + | | | Defense response |
|  |  |  | TraesCS3A02G479300 | 711040605 | | | | | | 711041129 | | + | | | Response to biotic stress |
|  |  |  | TraesCS3A02G479200 | 710946876 | | | | | | 710949891 | | + | | | Defense response and response to biotic stimulus |
|  |  |  | TraesCS3A02G479100 | 710935516 | | | | | | 710940717 | | + | | | Defense response |
|  |  |  | TraesCS3A02G478900 | 710837017 | | | | | | 710838334 | | + | | | Defense response |
| qSTB.18 | 3B | 17785833 | TraesCS3B02G035100 | 17081352 | | | | | | 17087675 | | - | | | Defense response |
|  |  |  | TraesCS3B02G035000 | 16983166 | | | | | | 16984121 | | + | | | Plant-type hypersensitive response. |
|  |  |  | TraesCS3B02G034700 | 16692513 | | | | | | 16694199 | | + | | | Plant-type hypersensitive response. |
|  |  |  | TraesCS3B02G034200 | 16333353 | | | | | | 16334691 | | + | | | Response to biotic stress. |
|  |  |  | TraesCS3B02G034000 | 16202219 | | | | | | 16202482 | | - | | | Response to biotic stress. |
|  |  |  | TraesCS3B02G033400 | 16078276 | | | | | | 16079730 | | - | | | Plant-type hypersensitive response. |
|  |  |  | TraesCS3B02G033400 | 16078276 | | | | | | 6079730 | | - | | | Regulation of plant-type hypersensitive response |
|  |  |  | TraesCS3B02G040100 | 19394360 | | | | | | 19396966 | | - | | | Defense response |
|  |  |  | TraesCS3B02G039200 | 18858691 | | | | | | 8865152 | | - | | | Defense response |
|  |  |  | TraesCS3B02G038600 | 18558602 | | | | | | 18559871 | | - | | | Response to biotic stress |
|  |  |  | TraesCS3B02G038500 | 18542363 | | | | | | 18542847 | | + | | | Defense response |
|  |  |  | TraesCS3B02G037700 | | 18306039 | | | | | 18306470 | | + | | | Defense response to fungi |
|  |  |  | TraesCS3B02G037900 | | 18393856 | | | | | 18394281 | | + | | | Defense response to fungi |
|  |  |  | TraesCS3B02G03750 | | 18243443 | | | | | 18248804 | | + | | | Defense response |
| qSTB.19 | 3B | 59645976 | TraesCS3B02G08140 | | 51244017 | | | | | 51245393 | | + | | | Defense response |
| qSTB.20 | 3D | 42679365 | TraesCS3D02G080600 | | 40526100 | | | | | 40531620 | | + | | | Response to biotic stress |
|  |  |  | TraesCS3D02G080000 | | 39921031 | | | | | 39931494 | | + | | | Response to biotic stress. |
|  |  |  | TraesCS3D02G088300 | | 45015144 | | | | | 45018965 | | + | | | Plant-type hypersensitive response. |
|  |  |  | TraesCS3D02G088100 | | | | | 44966972 | | 44967619 | | + | | | Defense response to fungi/regulation of plant-type hypersensitive response/systemic acquired resistance/ jasmonic acid mediated signaling pathway. |
| qSTB.21 | 3D | 593664469 | TraesCS3D02G509900 | | | | | 595062529 | | 595067402 | | - | | | Jasmonic acid mediated signaling pathway and defense response to bacterium. |
|  |  |  | TraesCS3D02G508900 | | | | | 594802578 | | 594803164 | | + | | | Response to stress. |
|  |  |  | TraesCS3D02G508700 | | | | | 594769919 | | 594770140 | | + | | | Any immune system process that functions in the calibrated response of an organism to a potential internal or invasive threat. |
|  |  |  | TraesCS3D02G508400 | | | | | 594573981 | | 594583032 | | + | | | Defense response |
|  |  |  | TraesCS3D02G506300 | | | | | 593235701 | | 593238423 | | + | | | defense response |
|  |  |  | TraesCS4A02G337400 | | | | | 619582627 | | 619584158 | |  | | | Response to biotic stress |
|  |  |  | TraesCS4A02G341300 | | | | | 621796765 | | 621806512 | | - | | | Regulation of plant-type hypersensitive response, systemic acquired resistance, defense response by callose deposition in cell wall and about induced systemic resistance. |
|  |  |  | TraesCS4A02G335500 | | | | | 618085607 | | 618089160 | | + | | | Defense response, physiological defense response, antimicrobial peptide activity, defense/immunity protein activity |
|  |  |  | TraesCS4A02G335600 | | | | | 618121544 | | 618125040 | | + | | | Defense response /immunity protein activity |
|  |  |  | TraesCS4A02G335700 | | | | | 618158574 | | 618175725 | | + | | | Defense response/immunity protein activity |
|  |  |  | TraesCS4A02G337700 | | | | | 619948780 | | 619950150 | | - | | | Response to biotic stress |
| qSTB.23 | 5A | 688359748 | TraesCS5A02G525600 | | | | | | 685875882 | 685876097 | | + | | | Defense response to fungi/resistance response to pathogenic fungi |
|  |  |  | TraesCS5A02G525500 | | | | | | 685869126 | 685870602 | | + | | | Regulation of defense response |
|  |  |  | TraesCS5A02G521400 | | | | | | 682617294 | 682617626 | | - | | | Regulation of non-apoptotic programmed cell death |
|  |  |  | TraesCS5A02G528100 | | | | | | 688297677 | 688302598 | | - | | | response to biotic stress. |
|  |  |  | TraesCS5A02G528000 | | | | | | 688292625 | 688295351 | | + | | | Plant-type hypersensitive response/ he rapid/localized death of plant cells in response to invasion by a pathogen/ immune response-regulating signaling pathway. |
|  |  |  | TraesCS5A02G527600 | | | | | | 688137629 | 688142671 | | - | | | Defense response to fungi |
|  |  |  | TraesCS5A02G526500 | | | | | | 687275788 | 687281990 | | - | | | Defense response/immunity protein activity |
| qSTB.24 | 5B | 487460716 | TraesCS5B02G299200 | | | | | | 482423553 | 482432342 | | - | | | Response to biotic stress. |
|  |  |  | TraesCS5B02G299000 | | | | | | 482288012 | 482291300 | | + | | | Defense response by callose deposition |
|  |  |  | TraesCS5B02G299000 | | | | | | 482288012 | 482291300 | | + | | | Defense response to fungi. |
|  |  |  | TraesCS5B02G302800 | | | | | | 487344161 | 487346328 | | - | | | Any immune system process that functions in the calibrated response of an organism to a potential internal or invasive threat |
| qSTB.25 | 5B | 538706298 | TraesCS5B02G356900 | | | | | | 536476323 | 536476925 | | + | | | Defense response/immunity protein activity |
|  |  |  | TraesCS5B02G359800 | | | | | | 539460745 | 539469556 | | - | | | MAPK cascade/systemic acquired resistance/salicylic acid mediated signaling pathway/jasmonic acid mediated signaling pathway/defense response to fungus/regulation of plant-type hypersensitive response. |
|  |  |  | TraesCS5B02G356800 | | | | | | 536384212 | 536385111 | | + | | | defense response/immunity protein activity |
|  |  |  | TraesCS5B02G360300 | | | | | | 539933883 | 539935311 | | + | | | Response to stress. |
| qSTB.26 | 5D | 541603929 | TraesCS1A02G356800 | | | | | | 539754423 | 539758138 | | + | | | Defense response /immunity protein activity |
|  |  |  | TraesCS1A02G356600 | | | | | | 539720660 | 539724029 | | + | | | Response to biotic stress. |
|  |  |  | TraesCS1A02G355300 | | | | | | 537966441 | 537967220 | | - | | | Salicylic acid-dependent systemic resistance/defense response to bacterium |
|  |  |  | TraesCS1A02G354300 | | | | | | 537345702 | 537347810 | | - | | | Response to biotic stress. |
|  |  |  | TraesCS1A02G354000 | | | | | | 537347810 | 537083849 | | + | | | Defense response |
|  |  |  | TraesCS1A02G358100 | | | | | | 540191129 | 540192273 | | + | | | Response to stress. |
| qSTB.27 | 6A | 607427728-609480220 | TraesCS6A02G385200 | | | | | | 603135847 | 603136814 | | + | | | Respiratory system process. |
|  |  |  | TraesCS6A02G385000 | | | | | | 60309120 | 603095476 | | + | | | Immune response-regulating signaling pathway. |
|  |  |  | TraesCS6A02G385000 | | | | | | 603091205 | 603095476 | | + | | | Plant-type hypersensitive response/defense response to bacterium |
|  |  |  | TraesCS6A02G384900 | | | | | | 603080652 | 603085053 | | + | | | Immune response-regulating signaling pathway/plant-type hypersensitive response. |
|  |  |  | TraesCS6A02G384700 | | | | | | 603045257 | 603046126 | | + | | | Response to biotic stress |
|  |  |  | TraesCS6A02G389000 | | | | | | 604607404 | 604610875 | | - | | | Defense response to fungi/defense response to fungus, resistance response to pathogenic fungus/response to parasitic fungi/response to parasitic fungus |
|  |  |  | TraesCS6A02G391700 | | | | | | 606435973 | 606436281 | | - | | | defense response to fungus. |
|  |  |  | TraesCS6A02G391300 | | | | | | 606094973 | 606095368 | | + | | | Defense response to fungus/ defense response to bacterium. |
|  |  |  | TraesCS6A02G391100 | | | | | | 606035615 | 606041135 | | + | | | Response to biotic stress. |
|  |  |  | TraesCS6A02G390300 | | | | | | 605324997 | 605339666 | | + | | | Defense response to fungus/salicylic acid mediated signaling pathway |
|  |  |  | TraesCS6B02G436100 | | | | | | 703463862 | 703466298 | | - | | | Response to stress. |
|  |  |  | TraesCS6B02G431700 | | | | | | 700953964 | 700954293 | | - | | | Defense response to fungus/defense response to bacterium. |
| qSTB.28 | 6B | 708272196 | TraesCS6B02G430800 | | | | | | 698974724 | 698978209 | | + | | | Defense response to fungus and regulation of plant-type hypersensitive response. |
|  |  |  | TraesCS6B02G430000 | | | | | | 697752877 | 697760334 | | - | | | response to biotic stress. |
|  |  |  | TraesCS6B02G428600 | | | | | | 696636476 | 696641207 | | + | | | Defense response |
|  |  |  | TraesCS6B02G428500 | | | | | | 696429438 | 696482513 | | - | | | regulation of plant-type hypersensitive response/response to stress. |
|  |  |  | TraesCS6B02G428200 | | | | | | 696344464 | 696345417 | | - | | | Response to stress. |
|  |  |  | TraesCS6B02G428000 | | | | | | 696148391 | 696152567 | | - | | | Defense response. |
|  |  |  | TraesCS6B02G427100 | | | | | | 695809523 | 695811508 | | - | | | Response to fungus, response to stress. |
|  |  |  | TraesCS6B02G425300 | | | | | | 694340532 | .694342363 | | + | | | Innate immune response. |
|  |  |  | TraesCS6B02G424000 | | | | | | 693923562 | 693930572 | | + | | | immune response-regulating signaling pathway/ defense response to bacterium. |
|  |  |  | TraesCS6B02G424200 | | | | | | 693945124 | 693946318 | | + | | | response to stress. |
| qSTB.29 | 7A | 116530515 | TraesCS7A02G154900 | | | | | | 10786890 | 107870802 | | + | | | Jasmonic acid mediated signaling pathway/ethylene-activated signaling pathway regulation of plant-type hypersensitive response/defense response to bacterium.  Defense response to fungus /systemic acquired resistance, |
|  |  |  | TraesCS7A02G160700 | | | | | | 116774158 | 116775320 | | - | | | Response to fungus/ response to jasmonic acid, jasmonic acid biosynthetic process. |
|  |  |  | TraesCS7A02G159400 | | | | | | 115815406 | 115818288 | | - | | | Systemic acquired resistance. |
|  |  |  | TraesCS7A02G159300 | | | | | | 115803096 | 115807251 | | - | | | Defense response to fungus/ systemic acquired resistance/obsolete pathogen-associated molecular pattern dependent induction by symbiont of host innate immune response |
|  |  |  | TraesCS7A02G159200 | | | | | | 115502958 | 115504909 | | - | | | Defense response to fungus/systemic acquired resistance, salicylic acid mediated signaling pathway/jasmonic acid mediated signaling pathway/regulation of plant-type hypersensitive response |
|  |  |  | TraesCS7A02G158800 | | | | | | 114413214 | 114414446 | | - | | | Systemic acquired resistance |
|  |  |  | TraesCS7A02G158000 | | | | | | 112263526 | 112266127 | | + | | | Response to fungus, response to biotic stress. |
|  |  |  | TraesCS7A02G156900 | | | | | | 109825167 | 109826129 | | + | | | Defense response to fungus/regulation of plant-type hypersensitive response/ salicylic acid biosynthetic process/systemic acquired resistance/ Jasmonic acid mediated signaling pathway. |
| qSTB.30 | 7A | 690377106-691722567 | TraesCS7A02G502500 | | | | | | 691521202 | 691522644 | | - | | | Defense response to bacterium. |
|  |  |  | TraesCS7A02G501800 | | | | | | 691522644 | 691290122 | | - | | | Defense response to bacterium/Cellular response to stress. |
|  |  |  | TraesCS7A02G501100 | | | | | | 690929758 | 690930261 | | - | | | Response to biotic stress. |
|  |  |  | TraesCS7A02G501000 | | | | | | 690917988 | 690918491 | | - | | | Response biotic stress |
|  |  |  | TraesCS7A02G500100 | | | | | | 690202537 | 690206037 | | - | | | defense response/(R)-limonene synthase activity. response to jasmonic acid. |
| qSTB.31 | 7B | 686989852 | TraesCS7A02G49960 | | | | | | 689919674 | 689926140 | | - | | | Defense response. |
|  |  |  | TraesCS7A02G498600 | | | | | | 688993023 | 688999806 | | - | | | Defense response. |
|  |  |  | TraesCS7A02G498200 | | | | | | 688685229 | 688694372 | | - | | | Defense response to bacterium. |
|  |  |  | TraesCS7A02G496900 | | | | | | 686683155 | 686684065 | | + | | | SCF ubiquitin ligase complex. |
|  |  |  | TraesCS7A02G496200 | | | | | | 686155127 | 686162048 | | + | | | Response to fungus. |
|  |  |  | TraesCS7A02G495400 | | | | | | 685581322 | 685586908 | | + | | | Jasmonic acid biosynthetic process/ regulation of defense response. |
|  |  |  | TraesCS7A02G492900 | | | | | | 681785989 | 681791135 | | - | | | Defense response to fungus. |
|  |  |  | TraesCS7B02G413800 | | | | | | 681849784 | 681852313 | | + | | | Response to salicylic acid |
|  |  |  | TraesCS7B02G412900 | | | | | | 680540509 | 680541684 | | - | | | Response to biotic stress. |
|  |  |  | TraesCS7B02G412400 | | | | | | 680217732 | 680217971 | | - | | | Response to stress |
|  |  |  | TraesCS7B02G411500 | | | | | | 680066562 | 680071534 | | + | | | Positive regulation of programmed cell death/response to biotic stress. |
| qSTB.32 | 7D | 21667638-63471279 | TraesCS7D02G041100 | | | | | | 20573622 | 20578504 | | + | | | Defense response |
|  |  |  | TraesCS7D02G040900 | | | | | | 20547371 | 20549251 | | + | | | Defense response |
|  |  |  | TraesCS7D02G040700 | | | | | | 20442479 | 20463194 | | + | | | Defense response |
|  |  |  | TraesCS7D02G039500 | | | | | | 20057020 | 20069082 | | - | | | Defense response |
|  |  |  | TraesCS7D02G038200 | | | | | | 19404885 | 19410860 | | - | | | Systemic acquired resistance/regulation of plant-type hypersensitive response. |
|  |  |  | TraesCS7D02G038100 | | | | | | 19399535 | 19401331 | | + | | | Defense response. |
|  |  |  | TraesCS7D02G037800 | | | | | | 19207405 | 19209126 | | - | | | Methyl jasmonate methylesterase activity. |
|  |  |  | TraesCS7D02G037700 | | | | | | 19049652 | 19053785 | | + | | | Systemic acquired resistance. |
|  |  |  | TraesCS7D02G037400 | | | | | | 18926487 | 18927411 | | + | | | Immune response-regulating cell surface receptor signaling pathway/ defense response. |
|  |  |  | TraesCS7D02G035900 | | | | | | 18401249 | 18403992 | | - | | | Defense response. |
|  |  |  | TraesCS7D02G035600 | | | | | | 18244409 | 18246083 | | + | | | Defense response. |
| qSTB.33 | 7D | 528900507-531439751 | TraesCS7D02G411100 | | | | | | 529956128 | 529956538 | | - | | | Immune response. |
|  |  |  | TraesCS7D02G41070 | | | | | | 529772570 | 529772953 | | + | | | Defense response. |
|  |  |  | TraesCS7D02G410400 | | | | | | 529681686 | 529682069 | | - | | | Defense response. |
|  |  |  | TraesCS7D02G409800 | | | | | | 528891729 | 528896252 | | + | | | Defense response |
|  |  |  | TraesCS7D02G409600 | | | | | | 528130780 | 528133540 | | + | | | Systemic acquired resistance, jasmonic acid mediated signaling pathway, salicylic acid biosynthetic process , and  regulation of plant-type hypersensitive response and  defense response to fungus. |

^a^ High confident genes annotated from IWGSC CS RefSeq ver. 1.0 [IWGSC Annotation v2.1](https://wheat-urgi.versailles.inrae.fr/Seq-Repository/Annotations) available at https://wheat-urgi.versailles.inrae.fr/Seq-Repository/Annotations. Retrieved on May 24,2021.
