# Supplementary material for: Genome-Wide Association Study Reveals Novel Genetic Loci for Quantitative Resistance to Septoria Tritici Blotch in Wheat (Triticum aestivum L.)
Source: Front Plant Sci. 2021 Sep 24;12:671323. doi: 10.3389/fpls.2021.671323 (PMC8500178; doi:10.3389/fpls.2021.671323)
Supplement: Supplementary file 8 [file Presentation_1.PPTX]

## Slide 1
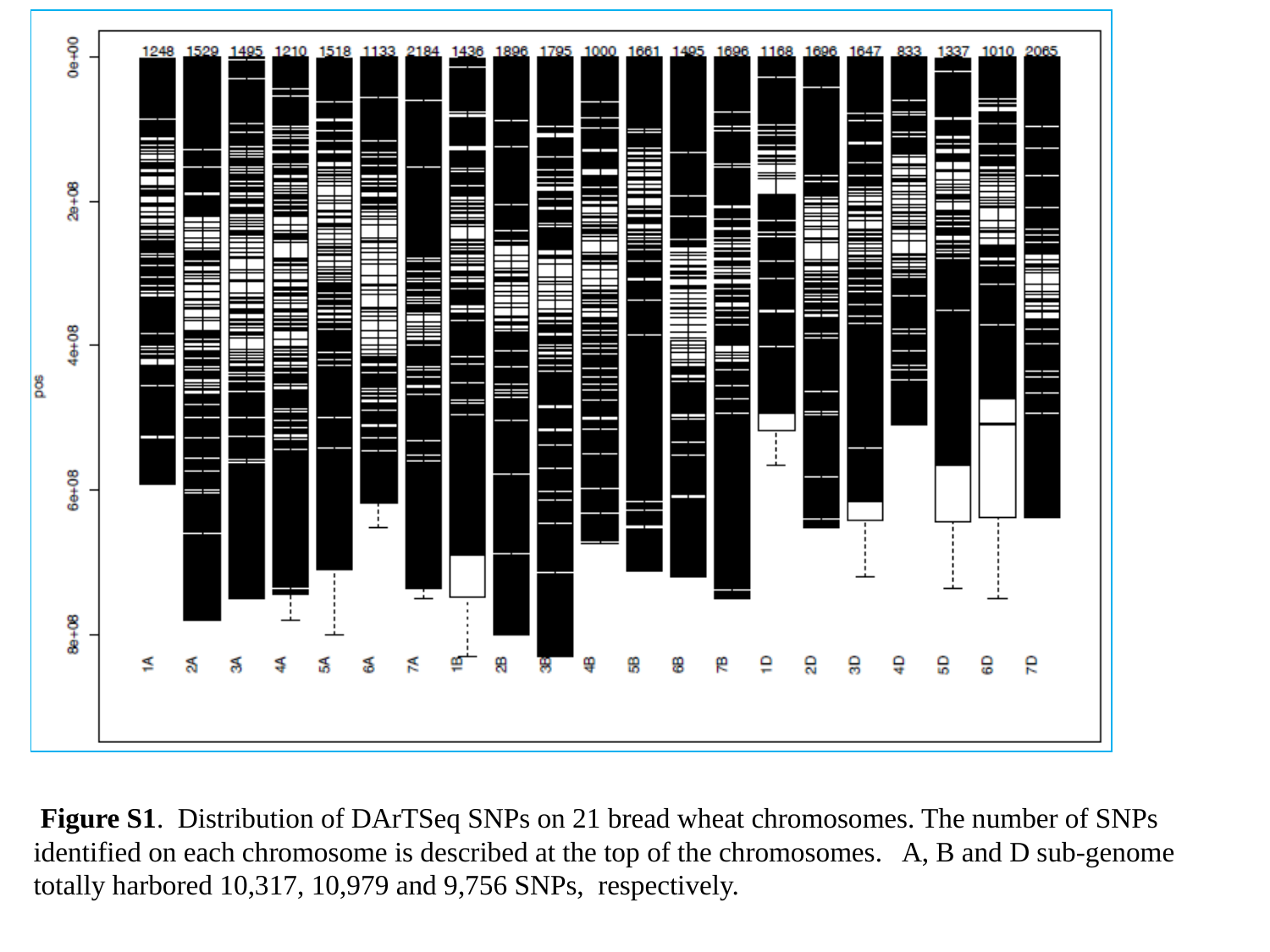

Figure S1. Distribution of DArTSeq SNPs on 21 bread wheat chromosomes. The number of SNPs identified on each chromosome is described at the top of the chromosomes. A, B and D sub-genome totally harbored 10,317, 10,979 and 9,756 SNPs, respectively.

## Slide 2
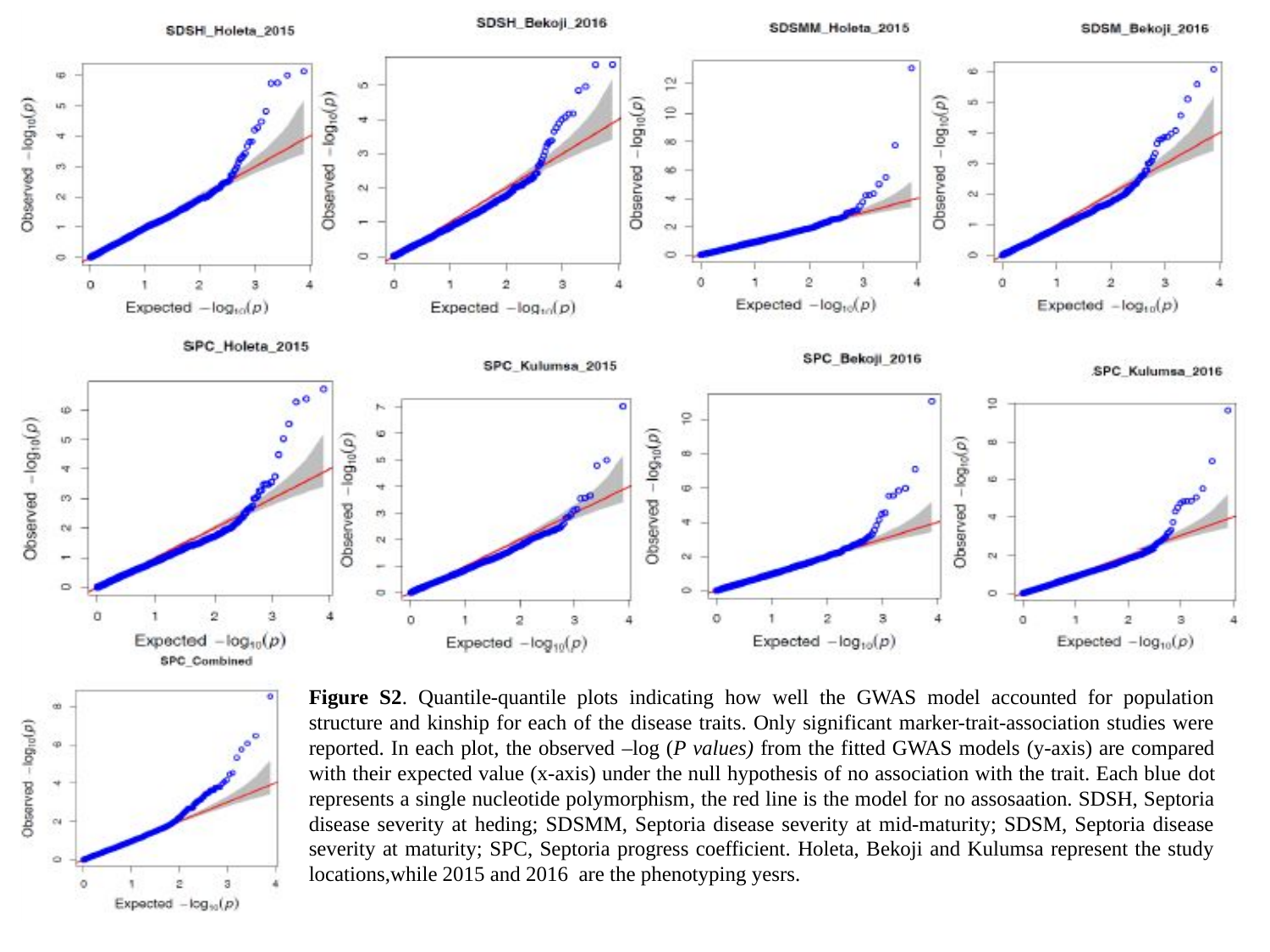

Figure S2. Quantile-quantile plots indicating how well the GWAS model accounted for population structure and kinship for each of the disease traits. Only significant marker-trait-association studies were reported. In each plot, the observed –log (P values) from the fitted GWAS models (y-axis) are compared with their expected value (x-axis) under the null hypothesis of no association with the trait. Each blue dot represents a single nucleotide polymorphism, the red line is the model for no assosaation. SDSH, Septoria disease severity at heding; SDSMM, Septoria disease severity at mid-maturity; SDSM, Septoria disease severity at maturity; SPC, Septoria progress coefficient. Holeta, Bekoji and Kulumsa represent the study locations,while 2015 and 2016 are the phenotyping yesrs.

## Slide 3
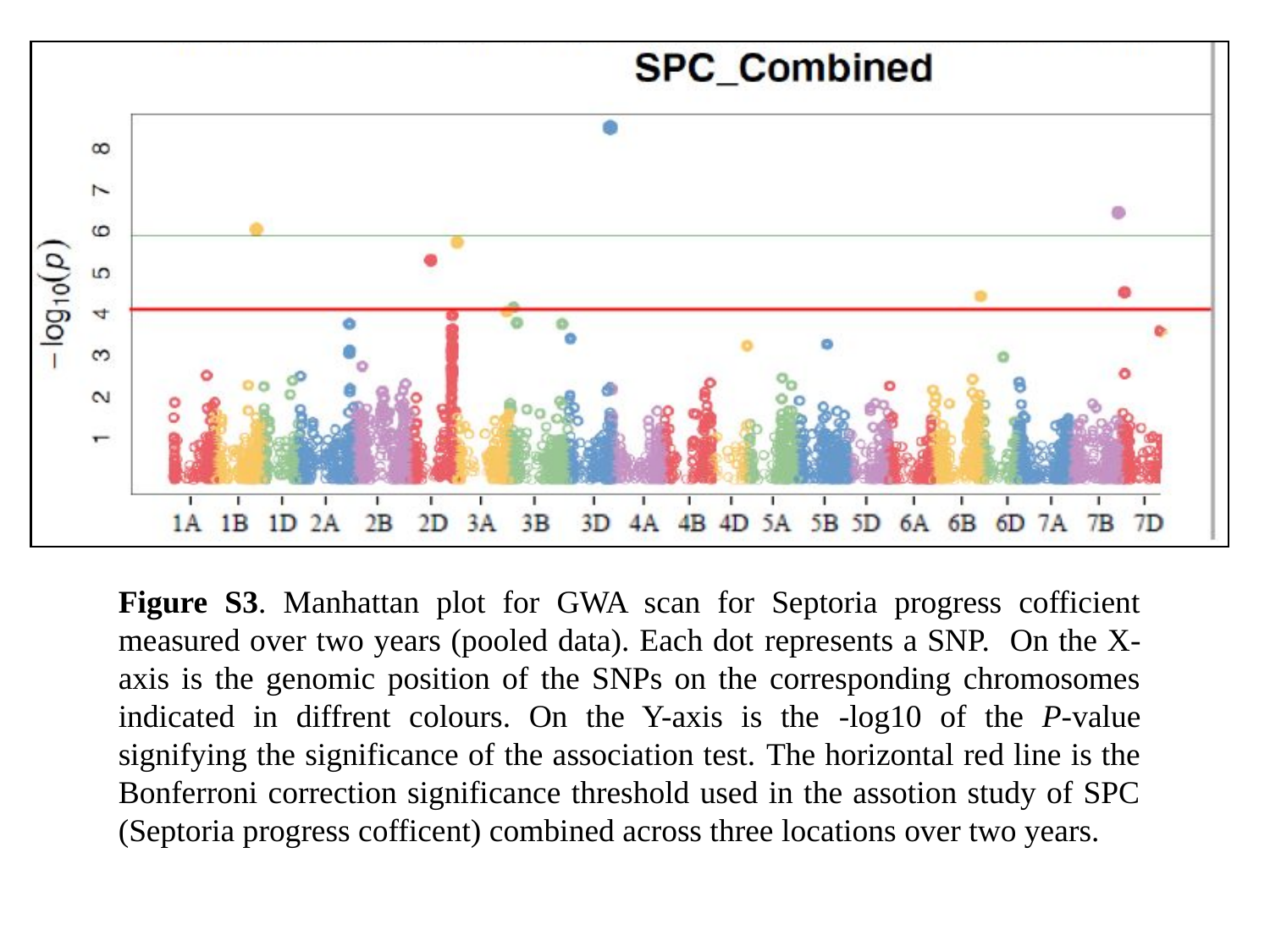

Figure S3. Manhattan plot for GWA scan for Septoria progress cofficient measured over two years (pooled data). Each dot represents a SNP. On the X-axis is the genomic position of the SNPs on the corresponding chromosomes indicated in diffrent colours. On the Y-axis is the -log10 of the P-value signifying the significance of the association test. The horizontal red line is the Bonferroni correction significance threshold used in the assotion study of SPC (Septoria progress cofficent) combined across three locations over two years.

## Slide 4
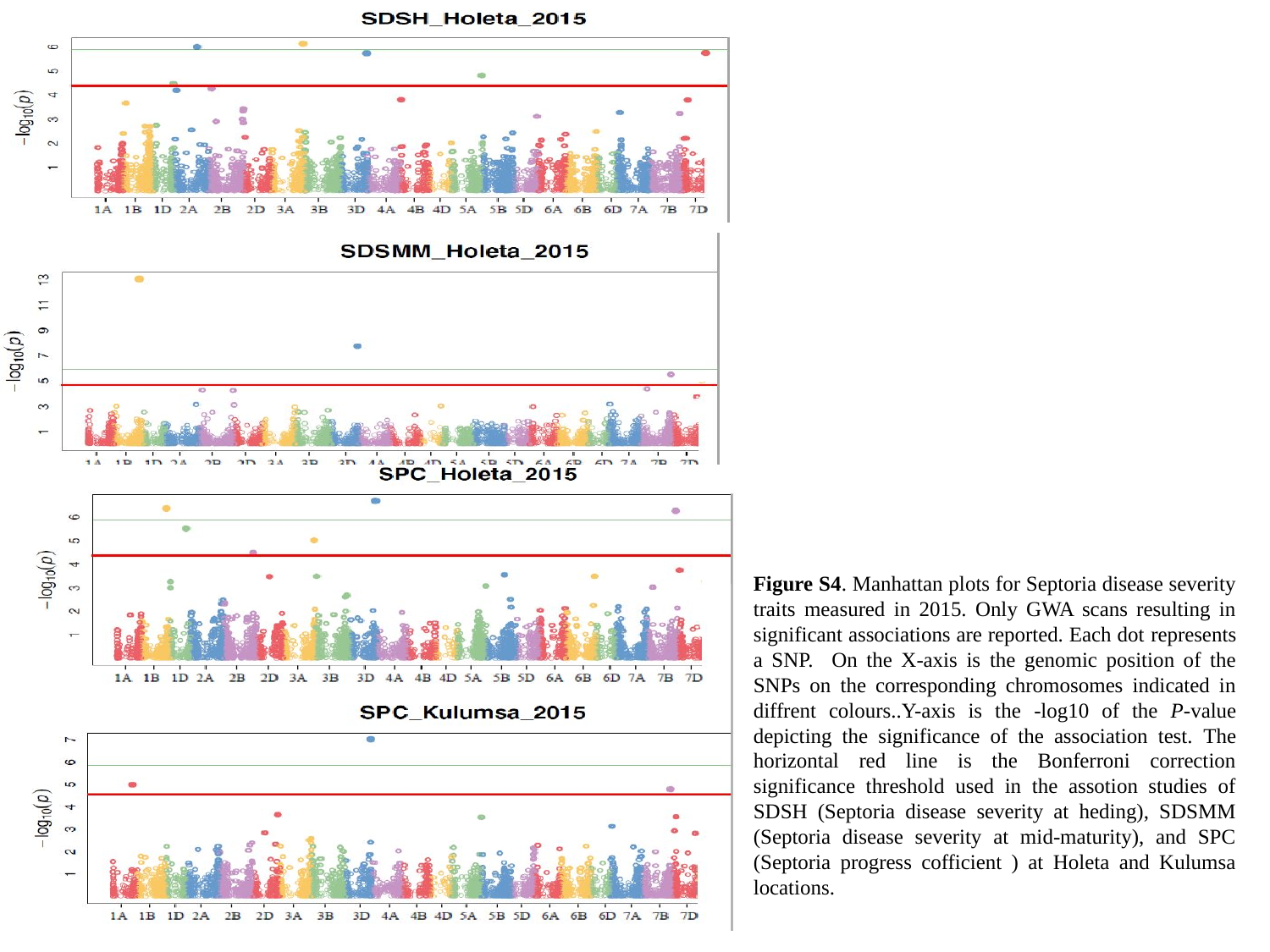

Figure S4. Manhattan plots for Septoria disease severity traits measured in 2015. Only GWA scans resulting in significant associations are reported. Each dot represents a SNP. On the X-axis is the genomic position of the SNPs on the corresponding chromosomes indicated in diffrent colours..Y-axis is the -log10 of the P-value depicting the significance of the association test. The horizontal red line is the Bonferroni correction significance threshold used in the assotion studies of SDSH (Septoria disease severity at heding), SDSMM (Septoria disease severity at mid-maturity), and SPC (Septoria progress cofficient ) at Holeta and Kulumsa locations.

## Slide 5
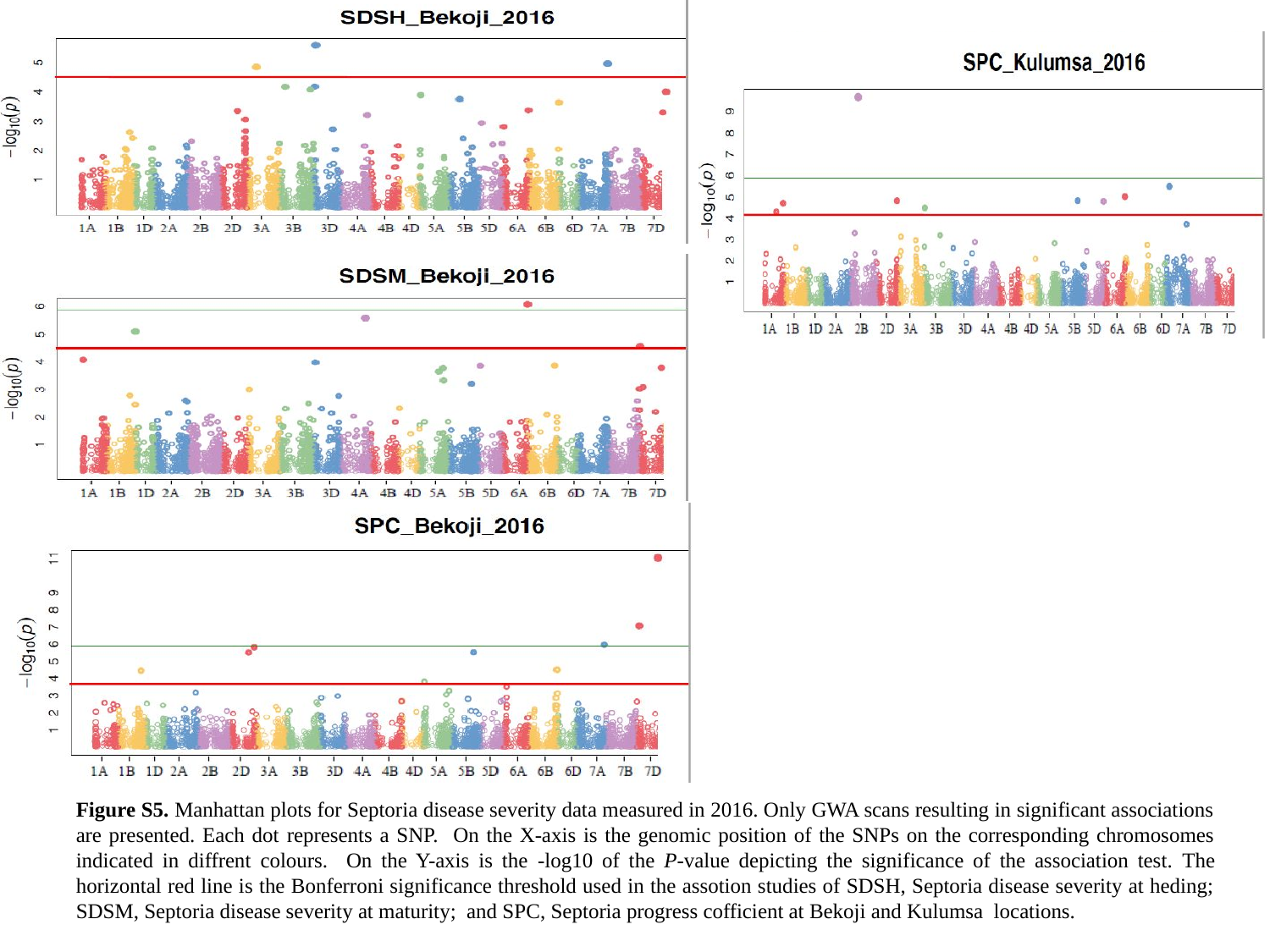

Figure S5. Manhattan plots for Septoria disease severity data measured in 2016. Only GWA scans resulting in significant associations are presented. Each dot represents a SNP. On the X-axis is the genomic position of the SNPs on the corresponding chromosomes indicated in diffrent colours. On the Y-axis is the -log10 of the P-value depicting the significance of the association test. The horizontal red line is the Bonferroni significance threshold used in the assotion studies of SDSH, Septoria disease severity at heding; SDSM, Septoria disease severity at maturity; and SPC, Septoria progress cofficient at Bekoji and Kulumsa locations.

## Slide 6
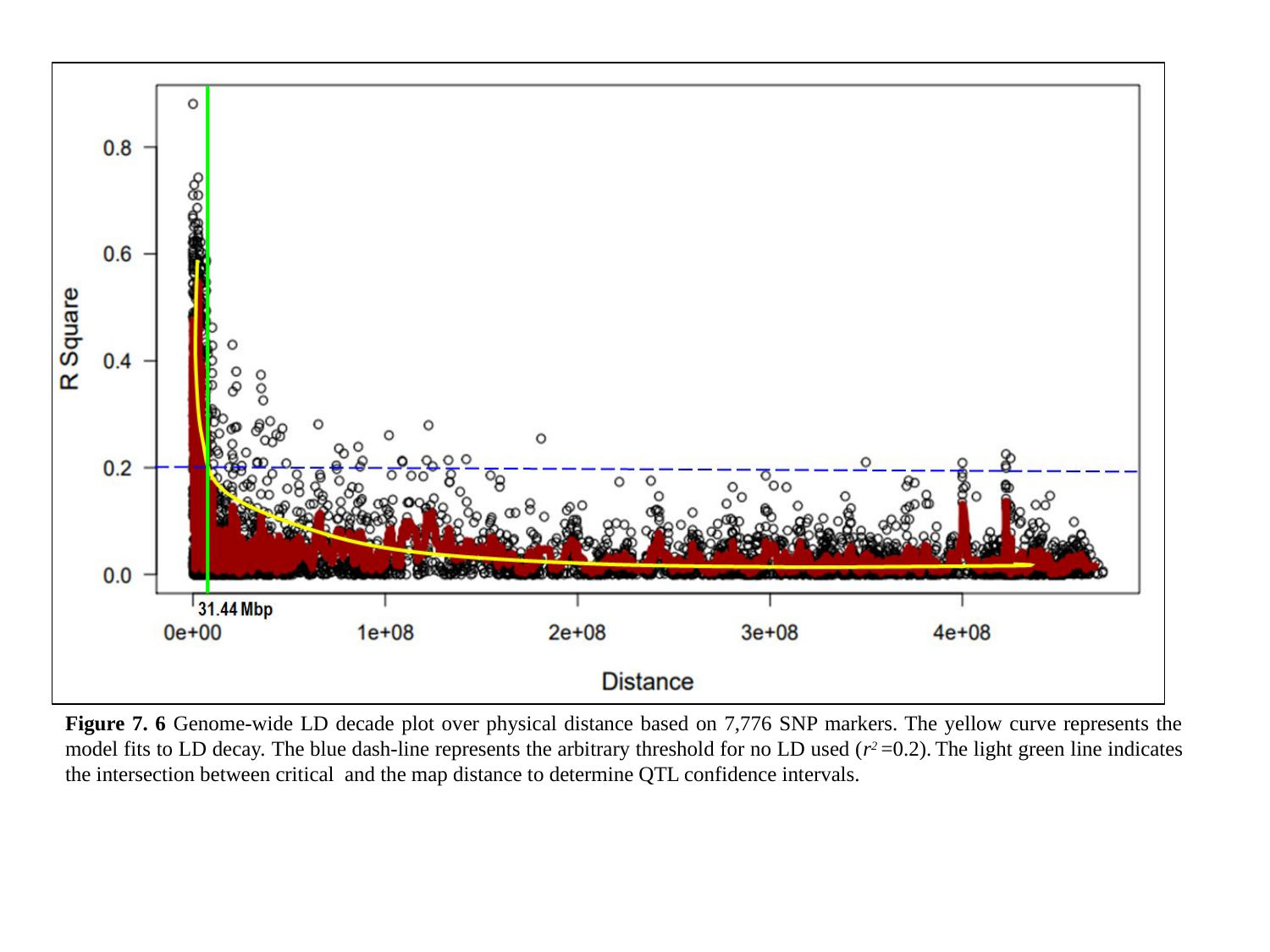

Figure 7. 6 Genome-wide LD decade plot over physical distance based on 7,776 SNP markers. The yellow curve represents the model fits to LD decay. The blue dash-line represents the arbitrary threshold for no LD used (r2 =0.2). The light green line indicates the intersection between critical and the map distance to determine QTL confidence intervals.

## Slide 7
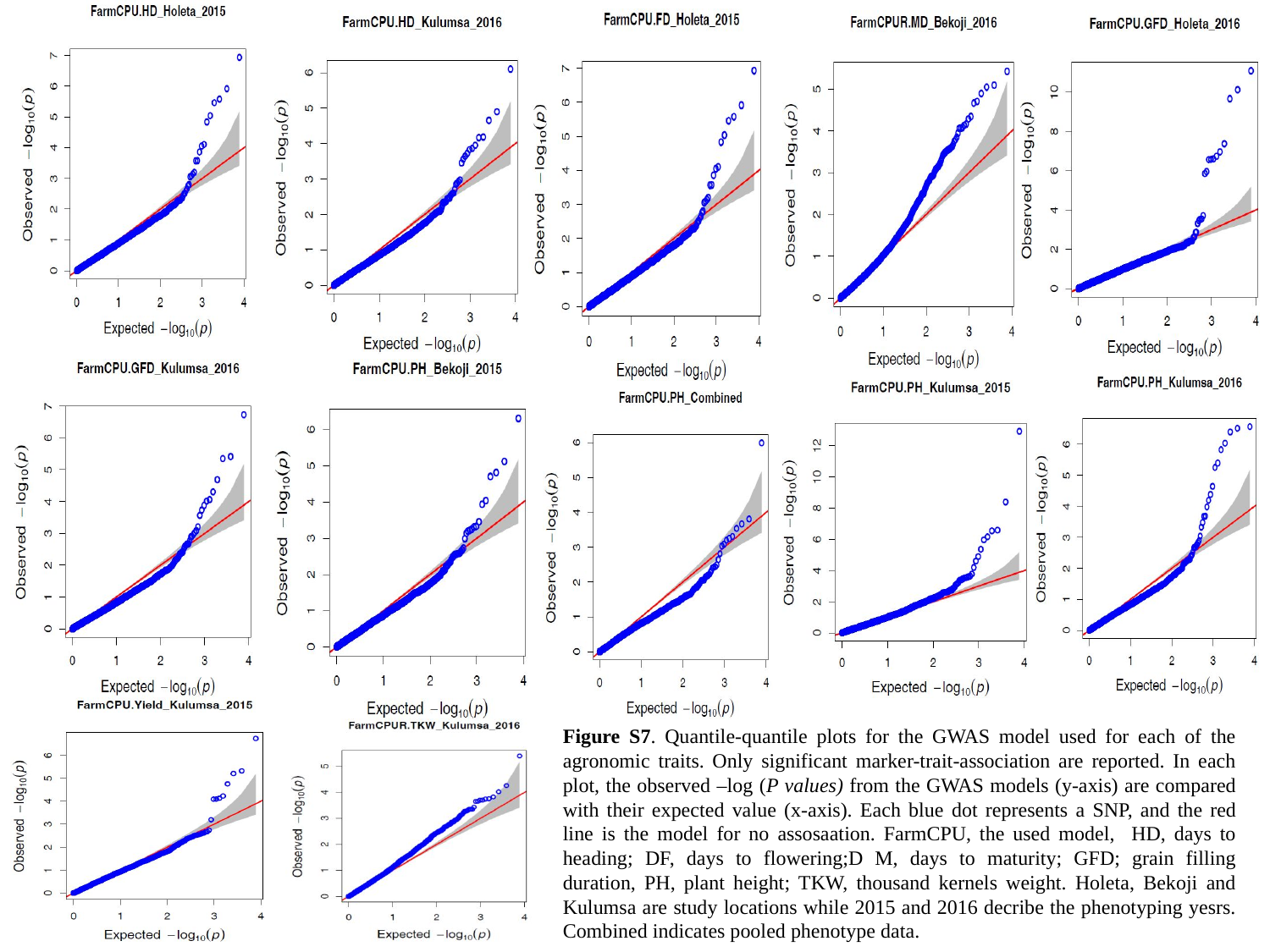

Figure S7. Quantile-quantile plots for the GWAS model used for each of the agronomic traits. Only significant marker-trait-association are reported. In each plot, the observed –log (P values) from the GWAS models (y-axis) are compared with their expected value (x-axis). Each blue dot represents a SNP, and the red line is the model for no assosaation. FarmCPU, the used model, HD, days to heading; DF, days to flowering;D M, days to maturity; GFD; grain filling duration, PH, plant height; TKW, thousand kernels weight. Holeta, Bekoji and Kulumsa are study locations while 2015 and 2016 decribe the phenotyping yesrs. Combined indicates pooled phenotype data.

## Slide 8
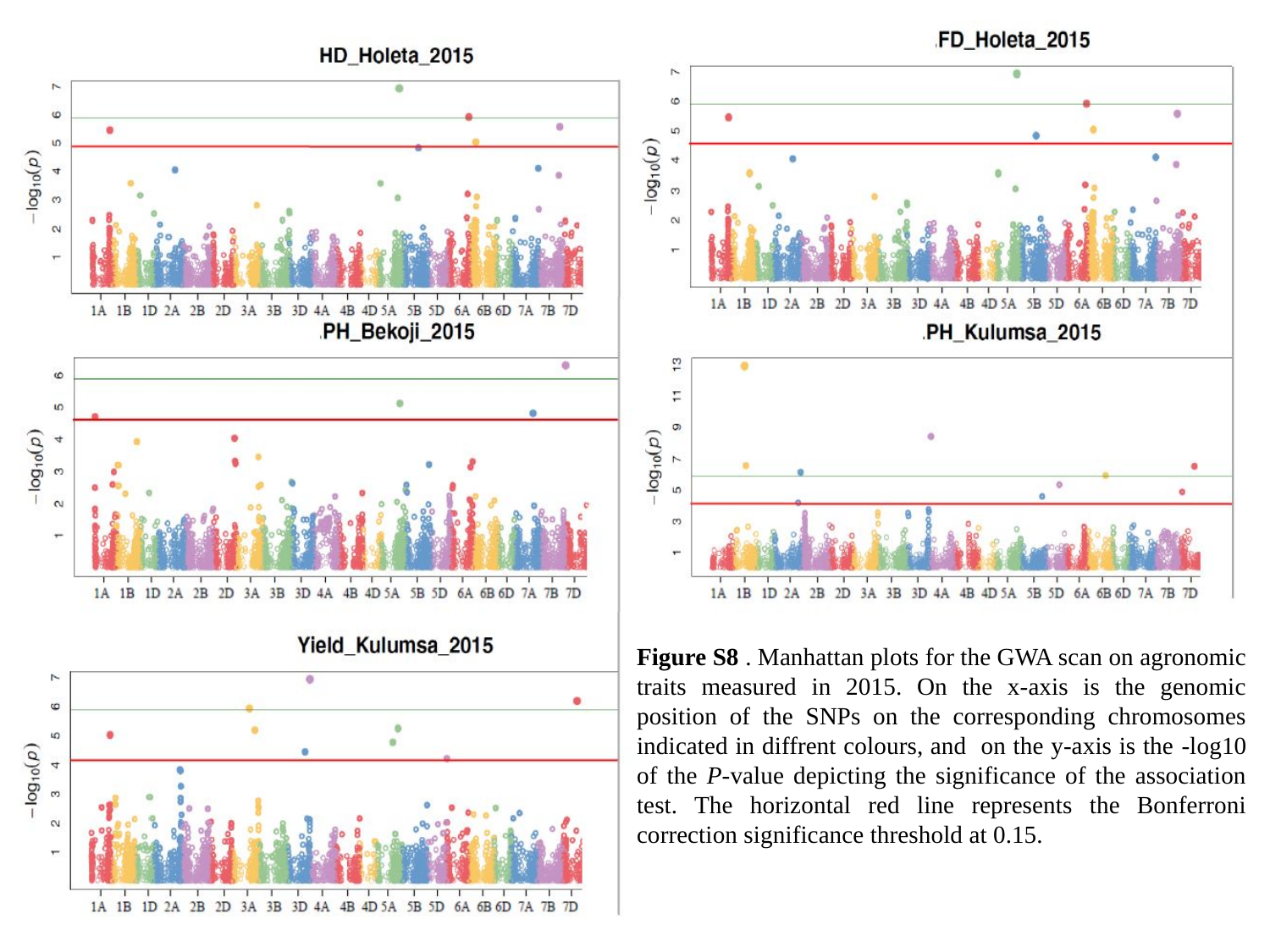

Figure S8 . Manhattan plots for the GWA scan on agronomic traits measured in 2015. On the x-axis is the genomic position of the SNPs on the corresponding chromosomes indicated in diffrent colours, and on the y-axis is the -log10 of the P-value depicting the significance of the association test. The horizontal red line represents the Bonferroni correction significance threshold at 0.15.

## Slide 9
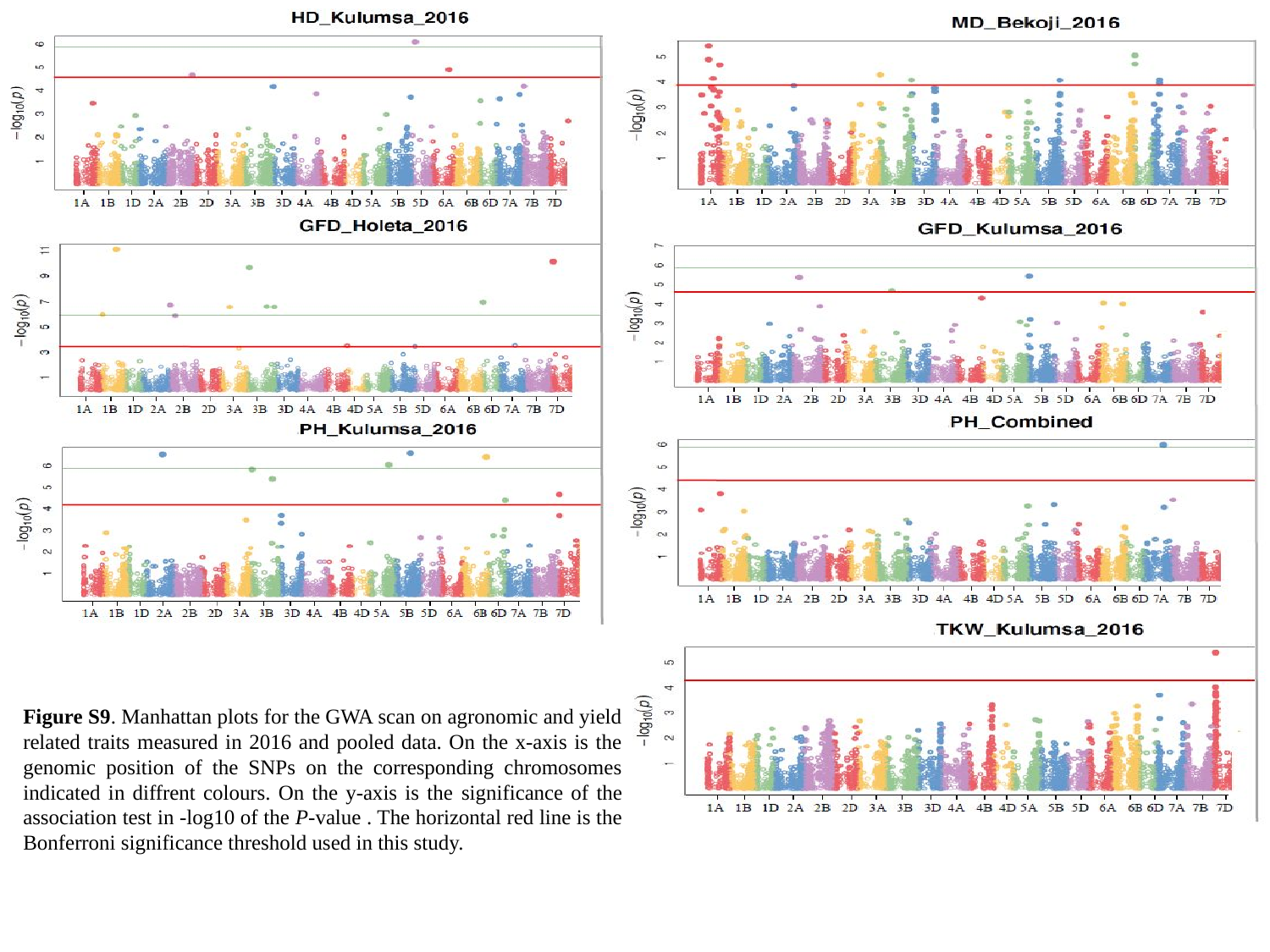

Figure S9. Manhattan plots for the GWA scan on agronomic and yield related traits measured in 2016 and pooled data. On the x-axis is the genomic position of the SNPs on the corresponding chromosomes indicated in diffrent colours. On the y-axis is the significance of the association test in -log10 of the P-value . The horizontal red line is the Bonferroni significance threshold used in this study.
